# ALDH1A3 Inhibition by *Prosopis farcta* Phytochemicals: A Computational Exploration of a Potential Antidiabetic Mechanism

**DOI:** 10.5812/ijpr-169211

**Published:** 2026-08-09

**Authors:** Nilufar Torabi, Gholamreza Taheripak, Nabaa Najjar, Mohammad Amin Nooraniyan Esfehani, Najme Sadat Hoseini, Ghahraman Abedi-Firouzjaei

**Affiliations:** 1 Department of Biochemistry, School of Medicine, Iran University of Medical Sciences, Tehran, Iran; 2 Department of Virology, School of Public Health, Tehran University of Medical Sciences, Tehran, Iran

**Keywords:** ALDH1A3 Inhibition, *Prosopis farcta*, Computational Pharmacology, Diabetes Mellitus, Molecular Dynamics, Phytochemicals, Molecular Docking

## Abstract

**Background:**

Diabetes mellitus is a chronic condition caused by insufficient insulin secretion or impaired insulin action. Traditionally, pancreatic β-cell loss has been attributed to hyperglycemia-induced apoptosis. However, more recent evidence indicates that β-cells can also undergo dedifferentiation, a process in which they lose their specialized identity and revert to a precursor-like state. ALDH1A3 has been reported as a marker associated with dedifferentiated β-cells in diabetes, making it a potential molecular target for studies of β-cell identity and their redifferentiation.

**Objectives:**

This study aimed to evaluate the potential interactions between phytochemicals in *Prosopis farcta* root extract and ALDH1A3 using an in silico workflow that included molecular docking, molecular dynamics simulations, and Molecular Mechanics Generalized Born Surface Area (MM-GBSA) analysis.

**Methods:**

This in silico study evaluated phytochemicals reported in the root extract of *Prosopis farcta*. The three-dimensional structures of the phytochemicals were downloaded from the PubChem database in SDF format. Molecular docking and molecular dynamics simulations were performed using PyRx and GROMACS, respectively. MM-GBSA analysis was performed using gmx_MMPBSA.

**Results:**

Docking analysis suggested that several *Prosopis farcta* phytochemicals could interact with functionally relevant regions of ALDH1A3, including the retinal- and NAD-binding regions. Among the evaluated compounds, luteolin 7-O-glucoside and luteolin 3′-glucoside exhibited favorable docking scores and stable interaction profiles in subsequent MD and MM-GBSA analyses. These findings suggest that these compounds may form energetically favorable interactions with ALDH1A3.

**Conclusions:**

Among the evaluated *Prosopis farcta* phytochemicals, luteolin 7-O-glucoside and luteolin 3′-glucoside emerged as the most promising computational candidates for further ALDH1A3-focused studies. These findings provide an initial basis for investigating whether interaction with ALDH1A3 may represent a potential mechanism contributing to the reported antidiabetic effects of *Prosopis farcta* extract and its possible relevance to β-cell dysfunction in diabetes. However, experimental validation is essential to confirm ALDH1A3 inhibition, its effects on β-cell redifferentiation, and antidiabetic potential.

## 1. Background

Diabetes mellitus is a chronic disease characterized by deficient insulin secretion or impaired insulin function. Diabetes complications include microvascular disorders, such as retinopathy, nephropathy, and neuropathy, and macrovascular disorders, such as coronary heart disease and cardiomyopathy (1, 2). The number of patients with diabetes was estimated at 463 million in 2019 and is expected to reach 700 million by 2045 (3). There are two main types of diabetes mellitus (DM): type 1 diabetes (T1DM), in which the immune system targets pancreatic cells, resulting in decreased insulin secretion, and type 2 diabetes (T2DM), in which insulin resistance occurs. As a result of reduced peripheral glucose uptake, pancreatic β-cells secrete more insulin; however, persistent hyperglycemia leads to oxidative stress in these cells, ultimately causing β-cell apoptosis (4, 5).

Prior research has shown that the loss of pancreatic β-cells in response to hyperglycemia is caused by apoptosis. However, recent studies suggest that, under certain pathological conditions, β-cells undergo a destructive process known as dedifferentiation. Furthermore, some β-cells can transdifferentiate into alpha or delta cells (6). This process results in the loss of the differentiated phenotype and cellular identity of pancreatic β-cells, converting them into precursor-like cells (7, 8). Inflammatory cytokines and hyperglycemia increase ER stress and oxidative stress, leading to the production of reactive oxygen species (ROS) in β-cells. This process impairs the function of essential proteins that play significant roles in insulin secretion and β-cell identity (9). Dedifferentiated β-cells show reduced expression of mature β-cell markers, such as PDX1, MAFA, insulin, NKX6.1, and FOXO1, while expressing proteins such as NGN3, OCT4, and ALDH1A3, which are associated with endocrine progenitor-like cells (10, 11). A process known as redifferentiation can convert dedifferentiated β-cells back to differentiated β-cells (12). With insulin therapy, anti-inflammatory treatments, and the use of proteins that enhance β-cell differentiation or inhibitors of proteins that induce β-cell dedifferentiation, β-cell redifferentiation can be achieved (13, 14).

Aldehyde dehydrogenase enzymes have 19 isoforms, and aldehyde dehydrogenase 1 isoform A3 (retinaldehyde dehydrogenase 3) acts in various cellular pathways, including the conversion of retinaldehyde to retinoic acid through NAD(P)-dependent irreversible oxidation. Retinoic acid can then bind to the retinoic acid receptor (RAR) and retinoid X receptor (RXR), which regulate cell proliferation and differentiation. ALDH1A3 also contributes to aldehyde detoxification and cellular responses to oxidative stress and has been investigated as a potential molecular target in several pathological conditions, including cancer (15). ALDH1A3 is a marker of pancreatic β-cell dedifferentiation and is related to T2D deterioration, but its mechanism is not well understood (16).

ALDH1A3 oxidizes aldehyde groups to generate NAD(P)H (17). Increased ALDH1A3 activity in pancreatic β-cells enhances their capacity to become progenitor-like cells in human and animal models, and it can be used as a biomarker for the diagnosis of β-cell dedifferentiation (16, 18, 19). ALDH1A3 is a tetrameric enzyme, and each monomer has independent catalytic functions. ALDH1A3 is composed of three functional domains: the NAD(P) ^+^ binding domain (L20–D149 and I171–G282), which contains a five-stranded parallel β-sheet; the catalytic domain (G283–M482), which has a six-stranded parallel β-sheet; and the oligomerization domain (K150–P170 and S483–L507), which features three-stranded antiparallel β-sheets (20). Trp189, Tyr472, and Cys314 play important roles in the ALDH1A3 catalytic domain. These residues may therefore be relevant to the design of compounds targeting functionally important regions of the enzyme (21).

Several studies have suggested that modulation or inhibition of ALDH1A3 may represent a potential strategy for investigating β-cell dedifferentiation in diabetes mellitus. Inhibition of ALDH1A3 restores β-cells by improving β-cell function and reversing their dedifferentiation (16, 18).

In addition, several studies have indicated that ALDH1A3 plays an important role in the development and progression of different cancers (22). Citral, a natural product, is a potent inhibitor of ALDH1A3 that binds to the substrate binding site and decreases the growth of breast cancer cells. DEAB (di-ethyl amino benzaldehyde), benomyl, benzyloxybenzaldehyde, and DIMATE (4-dimethylamino-4-methyl-pent2-ynthioic acid-S-methyl ester) inhibit the active site of all ALDH enzymes and show increased efficacy against ALDH1A3 (21, 23, 24).

*Prosopis farcta* extract contains flavonoid and polyphenol compounds that protect β-cells from oxidative stress both in vivo and in vitro; therefore, it reduces blood glucose and increases insulin production (25). A study conducted by Shridhar et al. evaluated rutin flavonoids against ALDH1A3 both in silico and in vitro. Their findings suggested that a rutin–copper complex exhibited anti-breast cancer activity associated with inhibition of ALDH1A3 (26). Given that rutin is one of the main phytochemicals present in *Prosopis farcta* extract, inhibition of ALDH1A3 and redifferentiation of β-cells may represent one possible mechanism underlying its effect in diabetes treatment.

## 2. Objectives

Therefore, this study aimed to evaluate potential interactions between phytochemicals reported in *Prosopis farcta* root extract and functionally relevant regions of ALDH1A3 using molecular docking, molecular dynamics simulations, and MM-GBSA analysis. It was designed to identify promising computational candidates for subsequent experimental investigation and to assess whether interactions with ALDH1A3 could provide a preliminary mechanistic hypothesis for some of the reported antidiabetic effects of *Prosopis farcta*.

## 3. Methods

### 3.1. Preparation of Ligands

Phytochemical compounds from *Prosopis farcta* root extract were identified through a literature review. For ligand preparation, three-dimensional structures of the phytochemicals were downloaded from the PubChem (27) database in SDF format. Energy minimization of the ligands was performed using PyRx-Python prescription 0.8 for 200 steps using the Universal Force Field (uff) and the conjugate gradient optimization algorithm (28).

### 3.2. Preparation of Protein

The crystal structure of ALDH1A3 (PDB ID: 5fhz) was retrieved from the Protein Data Bank (https://www.rcsb.org/) in PDB format (29). ALDH1A3 is a homotetramer composed of four structurally equivalent subunits, and each monomer contains an independent, structurally complete catalytic pocket comprising both substrate- and cofactor-binding sites. Therefore, one monomer was selected as a representative docking model to reduce redundancy and computational complexity. Monomer D was selected because of its well-resolved coordinates and catalytically relevant holo-like active-site conformation, which provided a complete representation of the catalytic tunnel without missing residues or structural ambiguities. The other monomers, heteroatoms, and water molecules were removed, and monomer D was saved separately in PDB format using YASARA software (http://www.yasara.org). Polar hydrogens and Kollman charges were then added using AutoDockTools 1.5.7 (30). Finally, energy minimization was performed using Swiss-PdbViewer (31).

### 3.3. Molecular Docking

To investigate interactions between compounds from the extract and ALDH1A3, molecular docking was performed using PyRx 0.8, which uses AutoDock Vina as its backend. The ligands and receptor were imported and converted to PDBQT format. To evaluate ligand interactions within structurally distinct regions of the catalytic tunnel, site-specific docking was performed using separate grid boxes for the substrate-binding and cofactor-binding pockets based on the crystallographic data for 5fhz. For the substrate-binding region (retinal site), the grid box was centered at X = 33.548, Y = 49.870, and Z = 67.915, with dimensions of 21 × 20 × 22 Å. For the cofactor-binding site (NAD^+^ site), the grid box was centered at X = 32.310, Y = 31.439, and Z = 72.647, with dimensions of 22 × 18 × 23 Å. Nine poses were generated for each ligand. The best pose was selected based on the docking score, binding orientation, and interactions with key catalytic residues. Binding interactions were analyzed and visualized using PyMOL 2.5.5, Discovery Studio, and Chimera software (32).

### 3.4. Docking Validation

Because the PDB structure used (5fhz) contains a naturally bound ligand and cofactor, the docking protocol was validated by removing and re-docking the co-crystallized retinoic acid and NAD into their respective binding sites. The re-docked complexes were superimposed on the corresponding reference crystallographic complexes using PyMOL, and root-mean-square deviation (RMSD) values were calculated. The docking protocol was considered valid and reliable when the re-docked ligand occupied a binding site and conformation similar to those of the co-crystallized ligand, with an RMSD ≤ 2 Å.

### 3.5. Molecular Dynamics

Molecular dynamics simulations of the protein–ligand complexes were conducted using GROMACS 2022.4 (33) for 100 ns. The CHARMM36 force field was used for parameterization, and energy files were written every 10 ps. The TIP3P water model was used for solvation, and the protein was centered in the simulation box with a minimum distance of 1 nm from the box edge. Energy minimization was performed for 10,000 steps using the steepest-descent algorithm. Equilibration was performed using the NVT and NPT ensembles for 50 ps (50,000 steps) each at 300 K and 1 bar, respectively. The production simulation was run for 50,000,000 steps with a 2-fs time step (100 ns) at 300 K and 1 bar using the velocity-rescaling thermostat and Parrinello-Rahman barostat, respectively. All bond lengths were constrained using the linear constraint solver (LINCS) algorithm. Periodic boundary conditions were applied in all directions. The Verlet scheme and particle-mesh Ewald (PME) method were used to calculate nonbonded and electrostatic interactions, respectively. Protein backbone RMSD, ligand RMSD, root-mean-square fluctuation (RMSF), hydrogen-bond occupancy, distances between each ligand and key residues, radius of gyration (Rg), and the number of contacts were calculated to analyze the trajectories (34).

### 3.6. MM-GBSA Analysis

MM-GBSA calculations were performed using gmx_MMPBSA version 1.6.2 to estimate the relative interaction energies of selected ALDH1A3–ligand complexes from MD trajectories (35). This post-MD energetic analysis evaluates ligand–protein interaction energies using conformations sampled during molecular dynamics simulations and therefore provides complementary energetic information beyond docking scores alone. For each complex, representative frames were extracted from the last 20 ns of the equilibrated MD trajectory, and binding-energy components, including van der Waals, electrostatic, polar solvation, and nonpolar solvation energies, were calculated. Final MM-GBSA values were reported as mean ± standard deviation.

## 4. Results

Several studies have reported the antidiabetic effects of *Prosopis farcta* in experimental models (25, 36). In parallel, increased ALDH1A3 expression has been associated with dedifferentiated pancreatic β-cells. These observations raise the possibility that modulation of ALDH1A3 may be relevant to β-cell identity and could represent one potential mechanism contributing to the reported antidiabetic effects of *Prosopis farcta* extract. Accordingly, the present study investigated the interactions of phytochemicals reported in *Prosopis farcta* extract with ALDH1A3 using molecular docking, molecular dynamics simulations, and MM-GBSA analysis. The aim was to identify promising computational candidates for further experimental evaluation in the context of ALDH1A3 modulation, β-cell dysfunction, and type 2 diabetes.

### 4.1. Molecular Docking

In the present study, a total of 20 main chemical compounds present in the *Prosopis farcta* extract were identified through a literature review. These ingredients include tannic acid, luteolin, 5-deoxy luteolin, apigenin, caffeic acid, isovitexin, kaempferol, myricetin, quercetin, rutin, naringenin, resveratrol, daidzein, luteolin 3'-glucoside, chlorogenic acid, myricetin 3-O-glucoside, luteolin 7-O-glucoside, quercetin 3-galactoside, quercetin 3-methyl ether, and vitexin. Each phytochemical was docked individually into two specific locations on ALDH1A3 monomer D: the substrate-binding pocket, which corresponds to the retinoic acid/retinal-binding site, and the cofactor-binding pocket, which corresponds to the NAD-binding site. Accordingly, two independent docking scores were obtained for each phytochemical, enabling its predicted interaction profile to be evaluated separately for each binding pocket. The resulting site-specific docking scores are presented in Table 1.

**Table 1.** Site-Specific Docking Scores of Retinal, NAD, and Phytochemicals Reported in *Prosopis farcta* Extract Within the Substrate- and Cofactor-Binding Pockets of ALDH1A3

Compound Name	PubChem ID	Docking score at the substrate-binding site (kcal/mol)	Docking score at the cofactor-binding site (kcal/mol)
**Retinal**	638015	-8.4	-
**NAD**	5893	-	-8.7
**Luteolin 7-O-glucoside**	5280637	-10.1	-9.0
**Luteolin 3'-glucoside**	12309350	-8.6	-9.6
**Myricetin**	5281672	-9.0	-8.4
**Isovitexin**	162350	-8.7	-8.2
**Rutin**	5280805	-9.0	-9.2
**Luteolin**	5280445	-8.8	-8.3
**Quercetin 3-O-galactoside**	5281643	-8.0	-8.6
**Chlorogenic Acid**	1794427	-8.6	-8.5
**5-deoxy luteolin**	5322065	-8.6	-8.7
**Apigenin**	5280443	-8.5	-8.1
**Myricetin 3-O-glucoside**	5318606	-8.7	-8.5
**Quercetin 3-methyl ether**	5280681	-8.4	-8.3
**Vitexin**	5280441	-8.9	-8.3
**Naringenin**	439246	-8.5	-8.1
**Kaempferol**	5280863	-8.4	-8.0
**Resveratrol**	445154	-8.3	-7.3
**Quercetin**	5280343	-8.5	-8.2
**Tannic acid**	17286569	-7.9	-9.2
**Daidzein**	5281708	-8.1	-7.5
**Caffeic acid**	689043	-6.8	-6.6

According to crystallographic data (20), retinoic acid, the enzyme product, occupies the active site in what has been described as a “substrate-like conformation.” The catalytic pocket of ALDH1A3 consists of a tunnel connecting the protein surface to the catalytic cysteine, which likely represents the pathway for substrate access and product release. This tunnel is formed by three α-helices and a surface loop. The catalytic tunnel begins at G136 at the entrance, continues through L471, and ends at the catalytic cysteine C314. Additional van der Waals contacts involve I132, G136, R139, T140, W189, L471, and A473.

**Figure 1.**
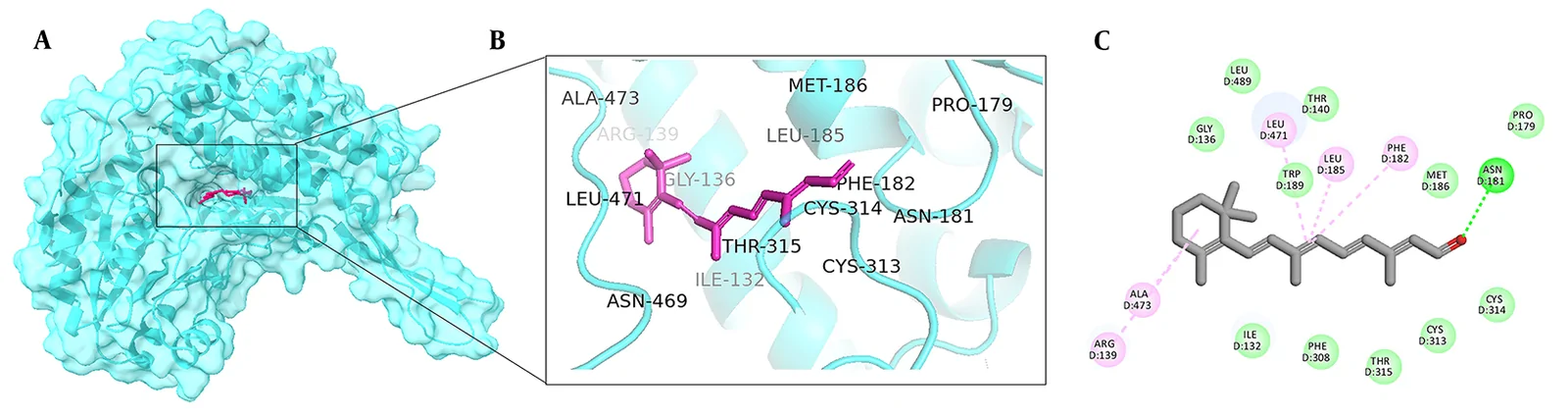
Retinal binding site. (A) Retinal position in the active site of ALDH1A3; (B) three-dimensional representation; and (C) two-dimensional interaction map.

As shown in Figure 1A, retinal was positioned within the substrate-binding region and interacted with several residues associated with the catalytic tunnel. Crystallographic data for retinoic acid indicate hydrogen bonds with M186, C314, and N181, whereas in our retinal docking pose, only N181 formed a hydrogen bond; interactions with M186 and C314 were mainly van der Waals. One possible explanation is that the crystallographic structure contains retinoic acid, the product, rather than retinal, the substrate. Carboxylic acids can form stronger and more numerous hydrogen bonds because they can act as both hydrogen-bond donors and acceptors, whereas aldehydes primarily act as hydrogen-bond acceptors and lack a hydroxyl group for donation (37). Despite these differences in interaction type, the docking pose placed retinal within the expected substrate-binding region, supporting the suitability of this pocket for subsequent site-specific comparisons of phytochemical poses (Figure 1A).

**Figure 2.**
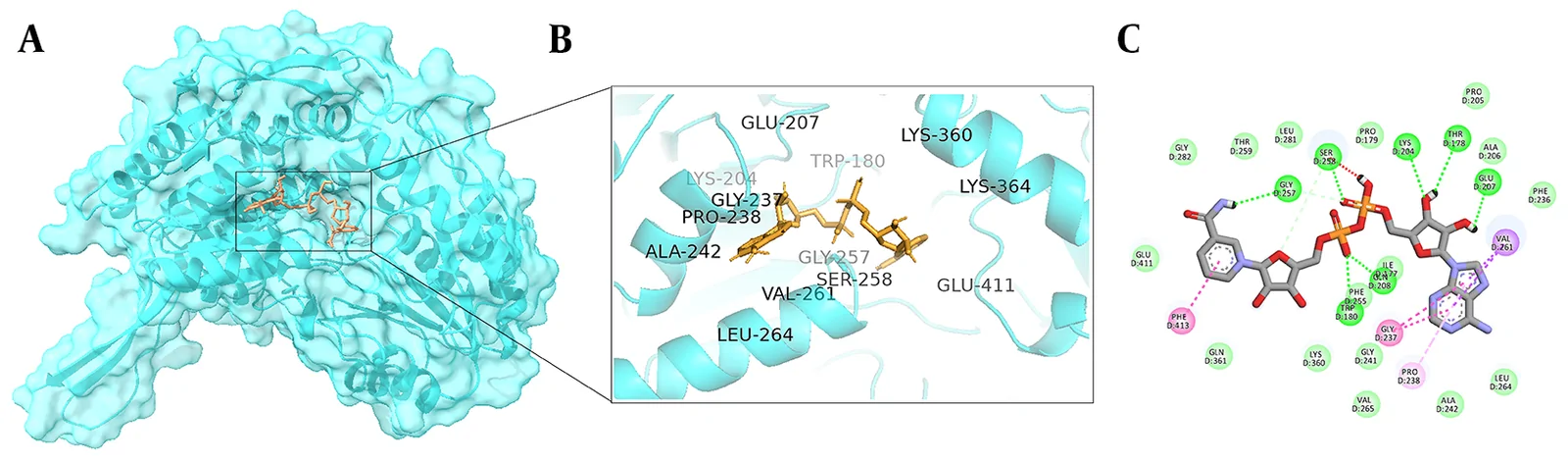
NAD binding site. (A) NAD position in the cofactor-binding site of ALDH1A3; (B) three-dimensional representation; and (C) two-dimensional interaction map.

As shown in Figure 2A, NAD was strongly bound and stabilized within the enzyme cofactor-binding site through numerous interactions. The adenine base of NAD formed van der Waals interactions with G237, P238, V261, A242, and L264. The adenine ribose formed hydrogen bonds with K204, T178, and E207 and van der Waals contacts with I177 and A206. The phosphate groups were stabilized by hydrogen bonds with W180, S258, and Q208 and by van der Waals interactions with P179 and F255; G257 formed a carbon–hydrogen bond. The nicotinamide ribose formed a van der Waals interaction with K360 and a carbon–hydrogen bond with S258. Finally, the nicotinamide group was stabilized by a hydrogen bond with G257 and van der Waals interactions with E411, T259, Q361, G282, and F413. These findings are generally consistent with ALDH1A3 crystallographic data, except for minor differences involving the nicotinamide ribose moiety (20). Notably, although the adenine ribose diphosphate moiety of NAD has a conserved location across ALDHs and enzyme monomers, the nicotinamide ribose moiety is variable and can adopt distinct conformations and interactions in different ALDHs and in different monomers of the same enzyme (38).

The site-specific docking results indicated that several phytochemicals could be accommodated within the substrate-binding pocket, the cofactor-binding pocket, or both predefined regions of ALDH1A3. Based on docking scores and predicted binding orientations, some compounds showed a relatively greater preference for the substrate-binding pocket, whereas others exhibited more favorable predicted interactions within the cofactor-binding pocket. Within each predefined binding site, various phytochemicals showed favorable docking scores compared with the corresponding reference ligand, indicating that they may adopt favorable predicted poses at functionally relevant sites of ALDH1A3. Although these ligand–receptor interactions suggest potential modulation/inhibition of ALDH1A3, docking studies alone do not provide evidence for inhibition of ALDH1A3 enzymatic activity. Because docking scores are approximate computational values and are affected by factors such as ligand size, polarity, conformational flexibility, and hydrogen-bonding capacity, they cannot be considered evidence of strong binding or enzyme inhibition. The predicted docking poses of the evaluated phytochemicals within the substrate-binding and cofactor-binding sites of ALDH1A3 are shown in Figure S1 in the Supplementary File.

**Figure 3.**
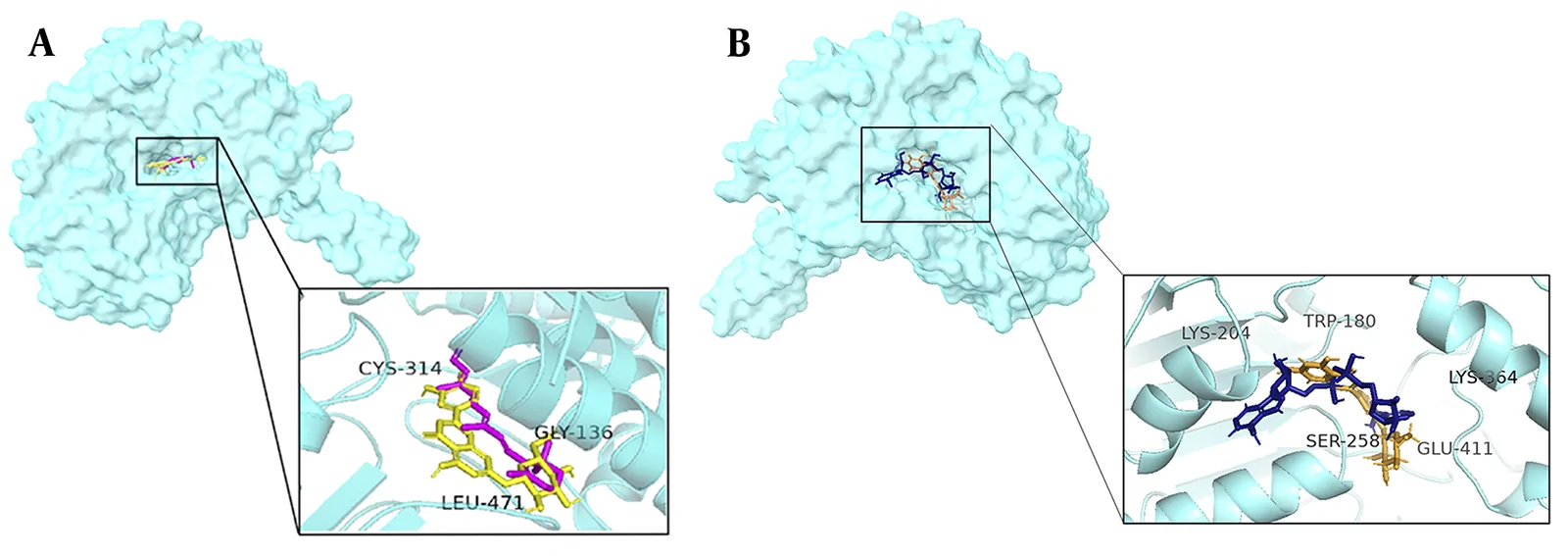
(A) Superimposed positions of luteolin 7-O-glucoside (yellow) and retinal (pink) within the substrate-binding pocket. (B) Superimposed positions of luteolin 3′-glucoside (orange) and NAD (navy blue) within the cofactor-binding pocket.

Within the substrate-binding pocket, luteolin 7-O-glucoside exhibited the most favorable docking score among the evaluated phytochemicals, with a value of −10.1 kcal/mol, whereas retinal showed a docking score of −8.4 kcal/mol. Luteolin 7-O-glucoside is a flavonoid present in *Prosopis farcta* branches and is recognized for a broad range of biological functions, particularly anti-inflammatory and antioxidant activities. Recent studies have also demonstrated its antiproliferative effects on human umbilical vein endothelial cells (HUVECs), indicating its potential role in inhibiting excessive cell growth associated with inflammation and tumorigenesis (39). Additionally, evidence suggests that luteolin and its derivatives have significant antidiabetic effects by lowering blood glucose levels, improving insulin sensitivity, reducing oxidative stress, and alleviating inflammation, thereby preventing the progression of diabetic complications (40-42). Ahmed et al. reported that luteolin 7-O-glucoside can effectively inhibit alpha-amylase and alpha-glucosidase activities, thereby contributing to lower blood glucose levels in patients with type 2 diabetes (43). Our docking findings suggest that ALDH1A3 inhibition may represent another possible molecular mechanism underlying these experimental observations, given the role of this enzyme in these pathways. According to Figure S1 in the Supplementary File, luteolin 7-O-glucoside forms hydrogen bonds with C314, W189, and R139, as well as van der Waals interactions with C313, M186, F182, F308, N469, L185, G136, L489, and I132. In addition, L471 is involved in both carbon–hydrogen bonding and van der Waals interactions in the ALDH1A3–luteolin 7-O-glucoside interaction. Notably, these close contacts correspond to those observed for retinal, the natural substrate of the enzyme. The most important contact is the hydrogen bond with C314, a key catalytic residue that is crucial for the conversion of retinal to retinoic acid by the enzyme (44). As mentioned previously, retinal is located in a catalytic pocket consisting mainly of G136, L471, and C314, together with additional van der Waals contacts. The predicted pose suggests that this ligand not only interacts with the catalytic cysteine but may also occupy much of the pocket by binding to these residues. Figure 3A illustrates the predicted spatial overlap between luteolin 7-O-glucoside and retinal within the substrate-binding region.

Within the cofactor-binding pocket, luteolin 3′-glucoside exhibited the most favorable docking score among the evaluated phytochemicals, with a value of −9.6 kcal/mol, compared with −8.7 kcal/mol for NAD. As shown in Figure S1 in the Supplementary File, W180, Q208, E260, G282, K364, E411, and Y437 are involved in hydrogen-bond formation with luteolin 3′-glucoside. In addition, it forms van der Waals interactions with T178, P179, A206, S258, L281, and G283, as well as a carbon–hydrogen bond with G257. Several of these residues are associated with the NAD-binding pocket. These findings suggest that luteolin 3′-glucoside can adopt a favorable predicted pose within the cofactor-binding region and interact with several residues involved in NAD recognition. Castellví et al. revealed that, in high-resolution crystal structures of ALDH1A3, ATP binds to the adenosine-binding pocket of the enzyme cofactor-binding site and inhibits its function by occupying several important residues involved in NAD binding (45). This observation supports the functional relevance of the cofactor-binding pocket as a potential target, although the effect of luteolin 3′-glucoside on NAD binding and ALDH1A3 activity requires experimental validation. Figure 3B highlights the predicted localization of luteolin 3′-glucoside within the NAD-associated cofactor-binding pocket.

The other phytochemicals reported in *Prosopis farcta* extract also showed favorable predicted interactions with ALDH1A3 in the site-specific docking analysis. Their relative docking preferences were evaluated by comparing docking scores and residue-level interactions within each predefined binding site. Some phytochemicals showed more favorable docking scores within the substrate-binding pocket, whereas others showed more favorable scores within the cofactor-binding pocket. Table 2 summarizes the major pocket-associated interactions of the selected phytochemicals and indicates the binding site in which each compound showed its more favorable or structurally relevant predicted interaction profile.

**Table 2.** Major Predicted Interactions and Preferred Binding-Site Assignments of Phytochemicals Reported in *Prosopis farcta* Extract Within ALDH1A3

Phytochemical	Binding Preference	Hydrogen Bond	van der Waals Interaction
**Luteolin 7-O-glucoside**	Substrate-binding site	C314, W189, R139	M186, L471, G136, I132
**Luteolin 3'-glucoside**	Cofactor-binding site	E260, K364	S258
**Myricetin**	Substrate-binding site	I132, L471	G136, M186
**Isovitexin**	Substrate-binding site	R139, C314, L471	I132, G136, N181, M186, W189
**Rutin**	Cofactor-binding site	S258	K204, V261
**Luteolin**	Substrate-binding site	-	I132, M186, W189, C314, L471
**Quercetin 3-O-galactoside**	Cofactor-binding site	S258	K204, V261
**Chlorogenic acid**	Substrate-binding site	A473	G136, C314, M186, L471
**5-deoxy luteolin**	Substrate-binding site	L471	I132, M186, W189, C314
**Apigenin**	Substrate-binding site	T315	I132, G136, M186, W189, L471
**Myricetin 3-O-glucoside**	Cofactor-binding site	Q361, Q208	V261, P238
**Quercetin 3-methyl ether**	Substrate-binding site	L471	M186, L471
**Vitexin**	Cofactor-binding site	W180, G257	Q361, S258, V261
**Naringenin**	Substrate-binding site	-	I132, G136, R139, M186, C314
**Kaempferol**	Substrate-binding site	N469, T315	M186, L471, W189, I132, C314
**Resveratrol**	Substrate-binding site	I132, C314	M186, L471, G136, N181
**Quercetin**	Cofactor-binding site	Q208, S258	G257, V261
**Tannic acid**	Substrate-binding site	L471, R139	I132
**Daidzein**	Cofactor-binding site	S258	K204, V261
**Caffeic acid**	Substrate-binding site	I132	L471, M186, G136

Some studies have shown that compounds located in the active site of ALDH1A3 can successfully inhibit enzyme function. Quattrini et al. showed that imidazo[1,2-a]pyridine derivatives could serve as novel ALDH1A3 inhibitors both in vitro and in silico. Their crystallographic and molecular modeling data revealed that the proposed compound is located in the catalytic site of the enzyme and interacts with C314, C315, F308, I321, W189, T140, R139, and N469. This compound showed a fully competitive inhibitory profile with a low IC50 value, indicating high potency against the enzyme in vitro. These compounds are promising candidates for treating cancers characterized by high ALDH1A3 expression (44). Additionally, Li et al. proposed MCI-INI-3 as a potent, selective competitive inhibitor of ALDH1A3 in mesenchymal glioma stem cells (GSCs). The compound occupies the enzyme active site, and ALDH1A3 inhibition, followed by a marked decrease in retinoic acid biosynthesis, impaired GSC proliferation and may provide a therapeutic strategy for gliomas (46). Similar studies have shown that ALDH1A3 inhibitors share a common interaction site with retinal, the natural substrate of the enzyme (21, 47). Collectively, these studies suggest that active-site occupation could be associated with ALDH1A3 inhibition when supported by biochemical, kinetic, or cell-based evidence.

Overall, the site-specific docking findings indicate that certain phytochemicals in *Prosopis farcta* extract could bind to functionally relevant ALDH1A3 regions, including the retinal- and NAD-binding sites. This observation provides an initial structural basis for investigating their effects on ALDH1A3 activity; however, experimental verification is required.

### 4.2. Docking Validation

The docking protocol was validated by re-docking retinoic acid into the substrate-binding pocket and NAD into the cofactor-binding pocket using the same docking parameters and site-specific grid boxes applied in the docking analysis. The resulting poses were superimposed on the corresponding crystallographic conformations in the ALDH1A3 structure (PDB ID: 5fhz). RMSD values of 0.85 Å for retinoic acid and 1.99 Å for NAD were obtained. Both values were within the predefined acceptance threshold of ≤ 2.0 Å, indicating that the docking protocol adequately reproduced the experimentally observed binding orientations at both sites (Figure S2 in the Supplementary File).

### 4.3. Molecular Dynamics Simulation

The two top-ranked phytochemicals identified by docking, together with the corresponding natural substrate/cofactor reference ligands, were selected for 100-ns molecular dynamics simulations. The simulated systems included apo ALDH1A3, ALDH1A3–luteolin 7-O-glucoside, ALDH1A3–luteolin 3′-glucoside, ALDH1A3–retinal, and ALDH1A3–NAD complexes. System behavior and stability were evaluated using backbone and ligand RMSD, Rg, ligand–protein contact number, ligand–residue distance analysis, RMSF, and hydrogen-bond occupancy.

**Figure 4.**
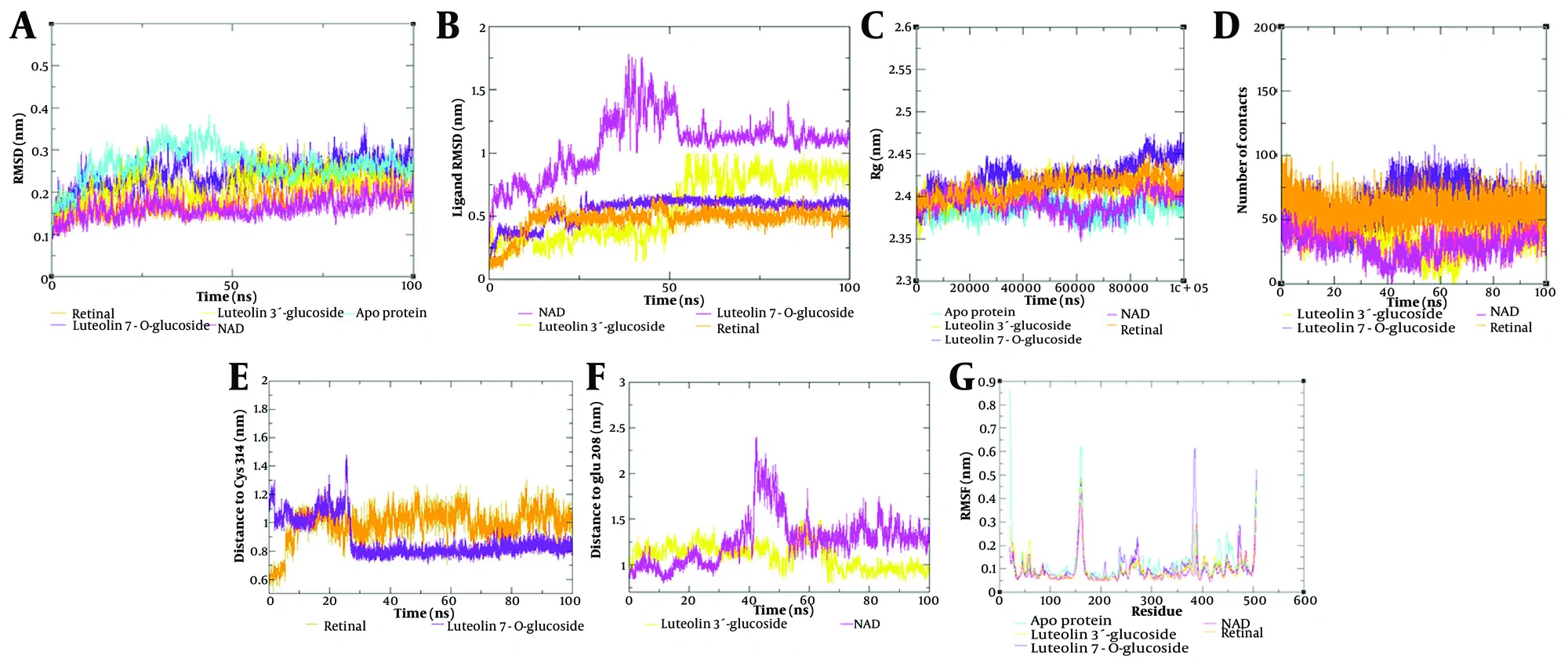
Molecular dynamics analyses of apo ALDH1A3 and selected ALDH1A3–ligand complexes during 100 ns simulations. (A) Backbone RMSD of apo ALDH1A3 and ALDH1A3–ligand complexes; (B) ligand RMSD profiles after protein backbone fitting; (C) radius of gyration analysis of apo ALDH1A3 and ALDH1A3–ligand complexes; (D) number of close ligand–protein contacts calculated using a 3 Å cutoff; (E) distance between substrate-site ligands and the catalytic residue Cys314; (F) distance between cofactor-site ligands and the NAD-associated residue Gln208; and (G) RMSF profiles of apo ALDH1A3 and ALDH1A3–ligand complexes.

Backbone RMSD was calculated to evaluate the overall structural stability of ALDH1A3 during the simulations. RMSD quantifies differences between the initial and subsequent atomic positions during MD. Figure 4A shows the RMSD of the ALDH1A3 backbone (monomer D) in the ligand complexes, with average RMSD (± SD) values of 0.26 (± 0.04) nm, 0.21 (± 0.03) nm, 0.24 (± 0.03) nm, 0.19 (± 0.04) nm, and 0.16 (± 0.02) nm for the apo protein, luteolin 3′-glucoside, luteolin 7-O-glucoside, retinal, and NAD, respectively. The relatively low RMSD values and reduced fluctuations after approximately 50 ns suggest that the protein backbone reached a stable conformational state in all simulated systems.

However, backbone RMSD reflects global protein stability and does not, by itself, establish ligand-binding stability. Ligand RMSD analysis was performed after fitting the trajectories to the protein backbone to evaluate ligand positional stability and reorientation within the predicted binding regions (Figure 4B). The ligand RMSD profiles showed different degrees of fluctuation among the complexes. Retinal displayed the lowest and most stable RMSD values, mainly fluctuating around 0.35 - 0.55 nm, suggesting limited positional deviation within the substrate-binding region. Luteolin 7-O-glucoside also remained comparatively stable, with RMSD values mostly around 0.50 - 0.65 nm after the initial equilibration phase. In contrast, luteolin 3′-glucoside increased after approximately 50 ns and subsequently stabilized at a higher RMSD plateau of about 0.75 - 0.95 nm, indicating possible reorientation within the cofactor-binding region. NAD exhibited the highest ligand RMSD variation, reaching approximately 1.6 - 1.8 nm during the middle phase of the simulation before stabilizing at a higher plateau of around 1.1 - 1.3 nm. This pattern suggests that the cofactor-site ligands underwent greater positional rearrangement than the substrate-site ligands. Therefore, ligand RMSD was not interpreted as a standalone indicator of binding stability. Instead, it was considered an initial descriptor of ligand positional fluctuation and reorientation, and its interpretation was further supported by the complementary analyses discussed below.

The radius of gyration (Rg) indicates the compactness of a protein structure. A larger Rg indicates a less compact protein–ligand complex. As shown in Figure 4C, the Rg profiles showed ligand-dependent variations during the simulation, indicating that ligand binding was associated with changes in the overall compactness of ALDH1A3. However, despite these fluctuations, the Rg profiles did not show a progressive increase or decrease throughout the trajectory, suggesting that no continuous unfolding, collapse, or major global destabilization occurred during the simulation period.

To estimate the number of contacts between the ligands and the protein, a maximum ligand–protein distance cutoff of 3 Å was applied and plotted throughout the simulation (Figure 4D). The average number of contacts between luteolin 7-O-glucoside (63.4 ± 13.8) and ALDH1A3 was greater than that between the protein and retinal (47.6 ± 18.1), and luteolin 3′-glucoside (36.9 ± 12.8) had more contacts with the protein than did NAD (32 ± 11.4).

Ligand–residue distance analysis was then performed to determine whether the observed ligand RMSD fluctuations corresponded to loss of binding-site localization or to local reorientation within the pocket. For the substrate-binding region, the distance between luteolin 7-O-glucoside or retinal and the catalytic residue Cys314 was monitored. As shown in Figure 4E, luteolin 7-O-glucoside initially showed a relatively greater distance from Cys314, followed by a marked decrease after approximately 25 ns and subsequent stabilization at a closer distance of approximately 0.75 - 0.85 nm. In contrast, retinal remained at a comparatively greater distance from Cys314 throughout most of the trajectory. This finding supports the interpretation that luteolin 7-O-glucoside underwent an early positional adjustment and then adopted a more Cys314-proximal orientation within the substrate-binding region.

For the cofactor-binding region, the distance between luteolin 3′-glucoside or NAD and the NAD-associated residue Gln208 was analyzed. As shown in Figure 4F, luteolin 3′-glucoside maintained a relatively stable distance from Gln208 during the simulation, whereas NAD displayed larger distance fluctuations, particularly during the middle phase of the trajectory. These results are consistent with the ligand RMSD profile, in which cofactor-site ligands showed greater positional rearrangement than substrate-site ligands. Importantly, the relatively stable Gln208-distance profile of luteolin 3′-glucoside, together with the later stabilization of NAD, indicates that the higher ligand RMSD values of the cofactor-site ligands did not necessarily reflect dissociation from the cofactor-binding pocket but more likely represented reorientation within or near the NAD-associated binding region.

RMSF measures the flexibility of each residue over the simulation period. It can also identify residues contributing to conformational deviations in the protein after ligand binding. Figure 4G shows that binding-site residues (110 - 136, 180 - 200, 300 - 320, and 430 - 471) fluctuated less in the ligand-bound complexes than in the apo protein, indicating lower mobility in these regions during the 100-ns simulations.

Hydrogen-bond occupancy analysis was performed to assess the persistence of polar ligand–protein interactions during the MD simulations (Table 3). At the cofactor-binding site, NAD formed persistent hydrogen bonds with E260, D358, and E207, with occupancies of 32.88%, 17.66%, and 15.31%, respectively. Luteolin 3′-glucoside formed hydrogen bonds with T256, W180, E207, and E411, with occupancies of 31.42%, 27.95%, 23.84%, and 15.20%, respectively. The shared hydrogen bond with E207 supports the association of luteolin 3′-glucoside with the NAD-related cofactor-binding site. At the substrate-binding site, retinal formed a hydrogen bond with C314 with 12.15% occupancy, whereas luteolin 7-O-glucoside showed higher occupancies with E280, L471, N469, and C314, at 68.97%, 42.84%, 37.34%, and 20.87%, respectively. The higher C314 occupancy of luteolin 7-O-glucoside compared with that of retinal supports its close and persistent positioning near the catalytic site. Hydrogen bonds with lower occupancy were regarded as transient and were not emphasized in the residue-level discussion. Overall, these results indicate that luteolin 7-O-glucoside and luteolin 3′-glucoside remained persistently associated with the substrate-binding and cofactor-binding regions of ALDH1A3, respectively.

**Table 3.** Hydrogen Bond Occupancy of Selected ALDH1A3–Ligand Complexes During Molecular Dynamics Simulations

System	H-Bond Residue	Occupancy (%)
**ALDH1A3-NAD**	E260	32.88
**ALDH1A3-NAD**	D358	17.66
**ALDH1A3-NAD**	E207	15.31
**ALDH1A3-luteolin 3′-glucoside**	T256	31.42
**ALDH1A3-luteolin 3′-glucoside**	W180	27.95
**ALDH1A3-luteolin 3′-glucoside**	E207	23.84
**ALDH1A3-luteolin 3′-glucoside**	E411	15.20
**ALDH1A3-Retinal**	C314	12.15
**ALDH1A3-Luteolin 7-O-glucoside**	E280	68.97
**ALDH1A3-Luteolin 7-O-glucoside**	L471	42.84
**ALDH1A3-Luteolin 7-O-glucoside**	N469	37.34
**ALDH1A3-Luteolin 7-O-glucoside**	C314	20.87

Representative trajectory frames extracted every 20 ns were used to visually assess ligand localization within the predicted binding regions (Figure S3 in the Supplementary File). The snapshots showed that all studied ligands remained within or near their corresponding binding pockets throughout the simulation. Luteolin 7-O-glucoside remained close to the substrate-binding site and retained proximity to residues C314, L471, N469, and R139, consistent with the ligand RMSD and hydrogen-bond occupancy analyses. Similarly, luteolin 3′-glucoside remained within the cofactor-associated site near W180, S258, E260, and E411. NAD remained within the NAD-binding pocket but showed local positional changes during the middle phase of the simulation, consistent with its higher ligand RMSD and distance fluctuations. Retinal remained within the substrate-binding pocket but at a greater distance from C314 than luteolin 7-O-glucoside. Overall, the representative snapshots support the interpretation that ligand RMSD fluctuations mainly reflected local reorientation or positional adjustment within the binding regions rather than complete ligand dissociation.

### 4.4. MM-GBSA Analysis

MM-GBSA is a widely used computational method in molecular modeling for estimating the free energy of binding between biomolecules, such as proteins and ligands, by combining molecular mechanics with implicit solvation models. In this study, MM-GBSA was used to complement docking and MD analyses by providing relative energetic estimates from MD-sampled conformations. This approach is useful because docking scores are calculated from static or semiflexible ligand–protein poses, whereas MM-GBSA incorporates conformational states obtained after dynamic relaxation of the complexes. The gmx_MMPBSA analysis was performed using the last 20 ns of the molecular dynamics simulations to estimate the relative interaction energies of the ALDH1A3–ligand complexes. In MM-GBSA analysis, ΔTOTAL represents the estimated total binding free energy of the ligand–protein complex, calculated from combined gas-phase molecular mechanics and solvation-energy contributions. More negative ΔTOTAL values generally indicate more favorable estimated ligand–protein interaction energies. The energy components calculated using gmx_MMPBSA are shown in Table 4. Luteolin 7-O-glucoside showed the most favorable average ΔTOTAL value among the analyzed complexes (-39.24 ± 3.66 kcal/mol), followed by retinal (-25.01 ± 2.29 kcal/mol), luteolin 3′-glucoside (-16.82 ± 3.09 kcal/mol), and NAD (-11.51 ± 3.41 kcal/mol).

**Table 4.** Detailed Energy Components of gmx_MMPBSA Analysis for Each Ligand During the Last 20 ns of Simulation

Ligand	ΔTOTAL (kcal/mol) (Average ± SD)	ΔVDWAALS (kcal/mol)	ΔEEL (kcal/mol)	ΔEGB (kcal/mol)	ΔESURF (kcal/mol)	ΔGGAS (kcal/mol)	ΔGSOLV (kcal/mol)
**Retinal**	-25.01 ± 2.29	-38.91	-3.19	22.48	-5.40	-42.10	17.08
**NAD**	-11.51 ± 3.41	-21.15	-432.05	446.18	-4.49	-453.20	441.69
**Luteolin 7-O-glucoside**	-39.24 ± 3.66	-43.63	-44.38	55.77	-6.99	-88.01	48.77
**Luteolin 3′-glucoside**	-16.82 ± 3.09	-30.70	-19.52	37.89	-4.49	-50.21	33.40

The MM-GBSA findings were consistent with the docking results and supported the observation that the selected luteolin glucosides can interact energetically with ALDH1A3. However, retinal, NAD, and flavonoid glucosides differ substantially in size, charge, polarity, and molecular composition; therefore, comparisons among their MM-GBSA values should be interpreted cautiously. These findings should be regarded as supportive energetic estimates rather than definitive evidence of binding affinity or competitive inhibition of ALDH1A3.

**Figure 5.**
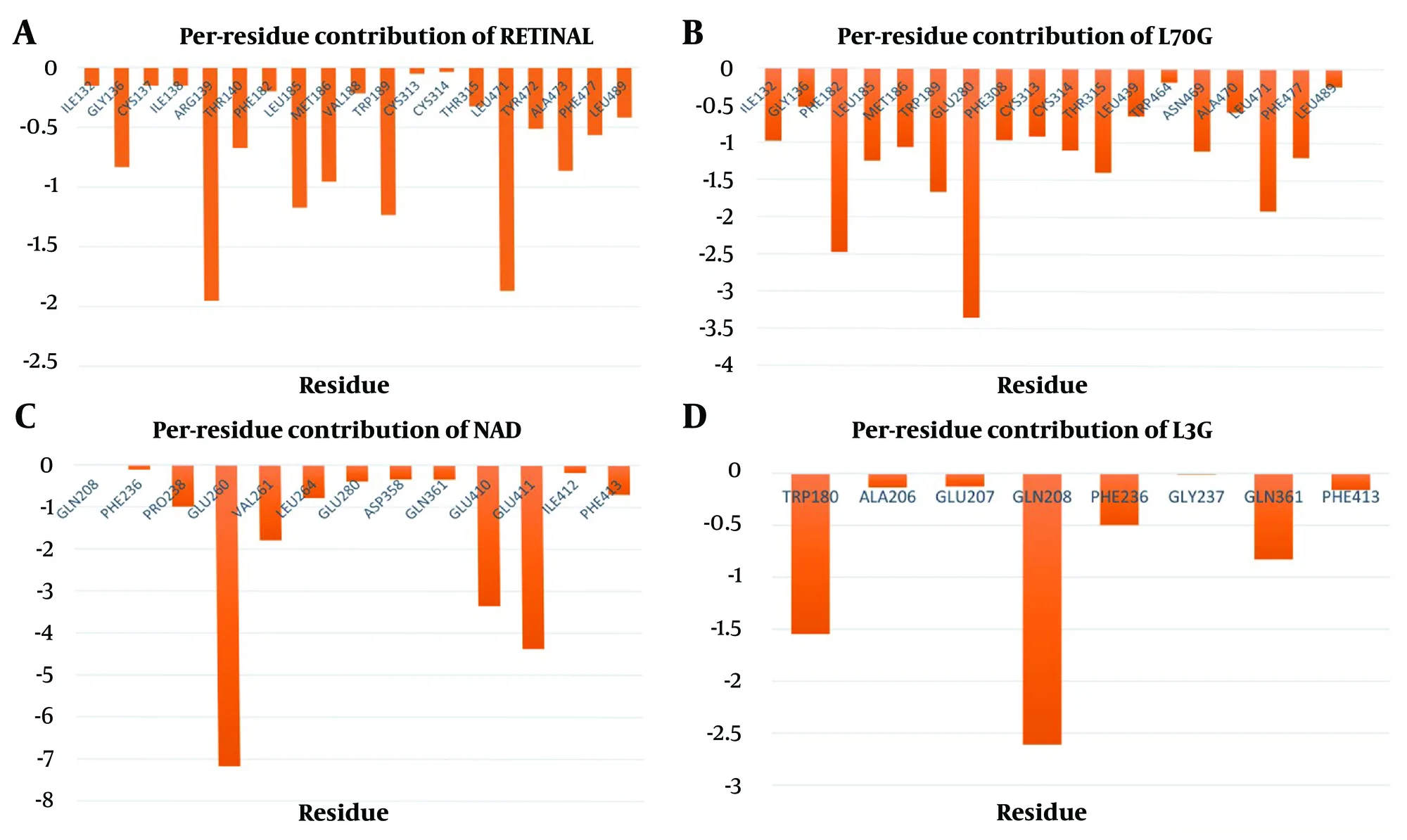
Per-residue free-energy contribution diagrams for amino acids involved in ligand–protein contacts during the last 20 ns of simulation for ALDH1A3 interactions with (A) retinal, (B) luteolin 7-O-glucoside, (C) NAD, and (D) luteolin 3′-glucoside.

Figure 5 shows per-residue free-energy contribution diagrams for amino acids involved in ligand–protein contacts during the last 20 ns of simulation. In this analysis, negative contribution values indicate residues with more favorable estimated energetic contributions to ligand interactions. For retinal–ALDH1A3, Arg139, Leu471, Trp189, Leu185, Met186, Ala473, and Gly136 were the major contributing residues. Luteolin 7-O-glucoside interacted with Glu280, Phe182, Leu471, Trp189, Thr315, Leu185, Phe477, Asn469, Cys314, and Met186. The involvement of several residues near the retinal-binding region, including Cys314, suggests that luteolin 7-O-glucoside may remain positioned within or close to a functionally relevant region of ALDH1A3 during the simulation. Residues contributing to the NAD–ALDH1A3 interaction included Gln208, Phe236, Pro238, Glu260, Val261, Leu264, Glu280, Asp358, Gln361, Glu410, Glu411, Ile412, and Phe413, whereas those associated with luteolin 3′-glucoside included Trp180, Ala206, Glu207, Gln208, Phe236, Gly237, Gln361, and Phe413. The overlap of several contributing residues with the NAD-binding region further supports the docking-based observation that luteolin 3′-glucoside may interact with residues associated with the cofactor-binding pocket.

## 5. Discussion

### 5.1. Limitations of the Study

Despite the use of integrated computational approaches, this in silico study has inherent limitations. Molecular docking and molecular dynamics simulations provide useful predictive insights but cannot fully reproduce the complexity of biological interactions in vivo.

Molecular docking provides predicted binding poses and docking scores; however, docking scores should not be interpreted as true binding affinities or as direct evidence of enzymatic inhibition because docking predicts binding and does not fully capture protein flexibility, solvent effects, or the dynamic nature of ligand–protein interactions (48, 49).

Although MD simulations provide valuable insights into structural stability and conformational behavior, the method is limited by computational cost, force-field accuracy, simulation timescale, system preparation, and sampling efficiency. Therefore, MD results should be interpreted as an approximation of system behavior rather than a complete representation (50, 51). Similarly, MM-GBSA calculations provide approximate relative binding-energy estimates and depend strongly on the quality of sampled MD snapshots and the selected implicit solvent model. Thus, MM-GBSA should be considered supportive rather than confirmatory evidence (52).

Another important limitation is that direct comparisons of docking scores among chemically distinct ligands, such as flavonoid glucosides, retinal, and NAD, should be interpreted with caution. These molecules differ markedly in size, polarity, charge distribution, flexibility, and hydrogen-bonding capacity. Therefore, more favorable docking scores for flavonoid compounds should not be considered direct evidence of a stronger true binding affinity or superiority over the natural substrate or cofactor. In this study, docking score comparisons were used only as preliminary, site-specific computational indicators and not as confirmatory evidence of enzymatic inhibition.

Finally, experimental validation is required to confirm the predicted interactions of *Prosopis farcta* phytochemicals with ALDH1A3 and to evaluate their biological and antidiabetic relevance.

### 5.2. Conclusions

ALDH1A3 has been characterized as a biomarker of pancreatic β-cell dedifferentiation, and its modulation has been investigated as an approach for studying β-cell identity and dysfunction in diabetes. In parallel, several studies have demonstrated the antidiabetic activity of *Prosopis farcta* extract in animal models. In this study, interactions between phytochemicals identified from *Prosopis farcta* extract and functional sites of ALDH1A3 were explored using molecular docking, molecular dynamics simulations, and MM-GBSA analysis. Several phytochemicals showed the potential to interact with either the substrate-binding or cofactor-binding site of ALDH1A3. Among the tested compounds, luteolin 7-O-glucoside and luteolin 3′-glucoside had the most favorable computational profiles for the substrate-binding and cofactor-binding sites, respectively. Subsequent MD and MM-GBSA analyses further supported favorable predicted interaction profiles for these compounds during the simulation period. These findings provide a preliminary structural and energetic basis for considering luteolin 7-O-glucoside and luteolin 3′-glucoside as computational candidates for further ALDH1A3-focused studies. The predicted interactions may represent one possible molecular hypothesis contributing to the reported biological effects of *Prosopis farcta* extract. Further enzymatic inhibition assays, β-cell-based experiments, and in vivo investigations are required to validate the biological relevance and potential antidiabetic effects of these phytochemicals.

ijpr-25-1-169211-s001.pdf

## Data Availability

The dataset presented in the study is available on request from the corresponding author during submission or after publication.
