# Supplementary material for: ALDH1A3 Inhibition by Prosopis farcta Phytochemicals: A Computational Exploration of a Potential Antidiabetic Mechanism
Source: Iran J Pharm Res. 2026 Aug 9;25(1):e169211. doi: 10.5812/ijpr-169211 (PMC13589463; doi:10.5812/ijpr-169211)
Supplement: Supplementary file 1 [file ijpr-25-1-169211-s001.pdf]

<table>
<tr><th>Ligand</th><th>Predicted position within the substrate-binding site</th><th>Predicted position within the cofactor-binding site</th></tr>
<tr><td>Luteolin 7-O-glucoside</td><td>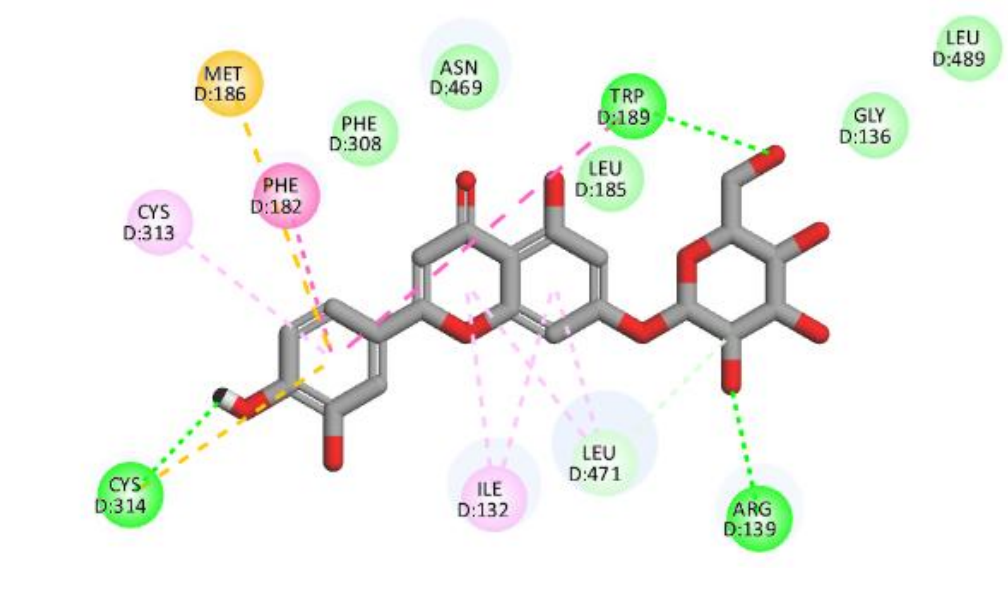</td><td>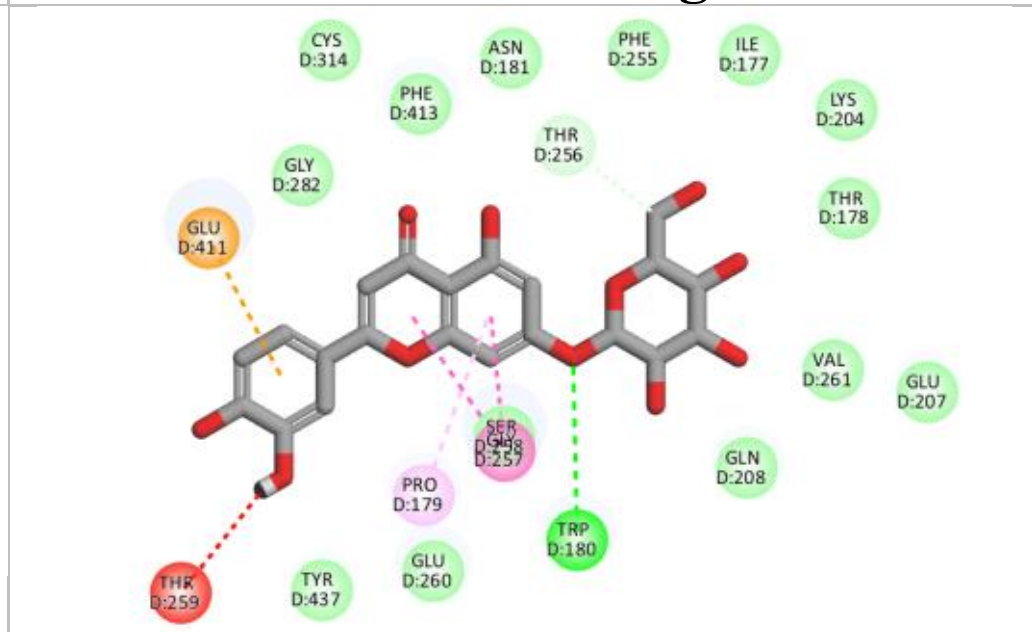</td></tr>
<tr><td>Luteolin 3'-glucoside</td><td>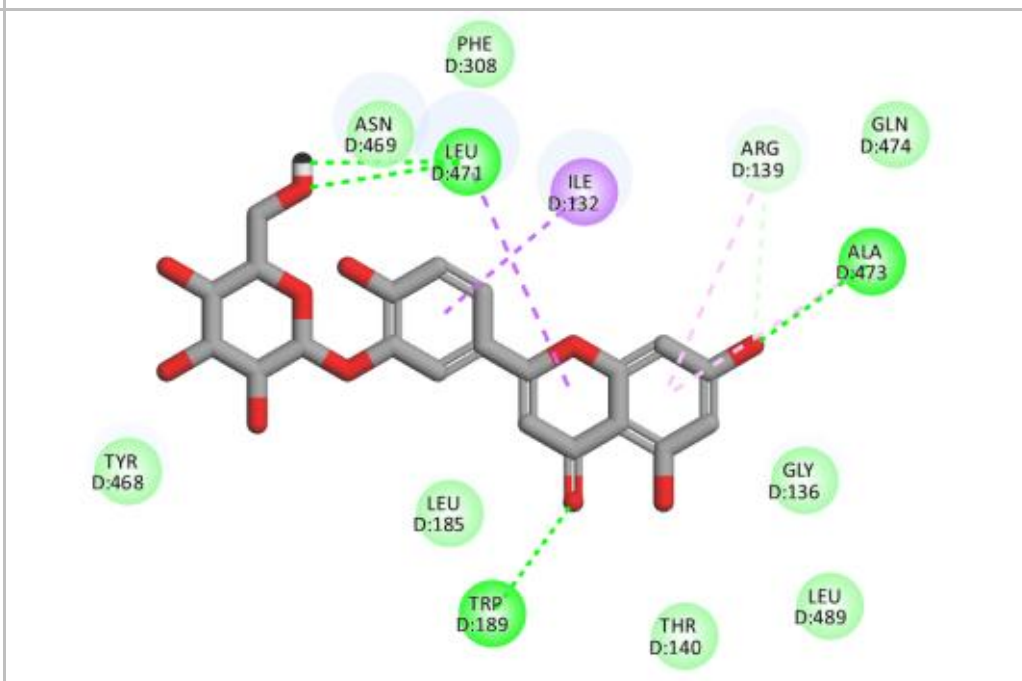</td><td>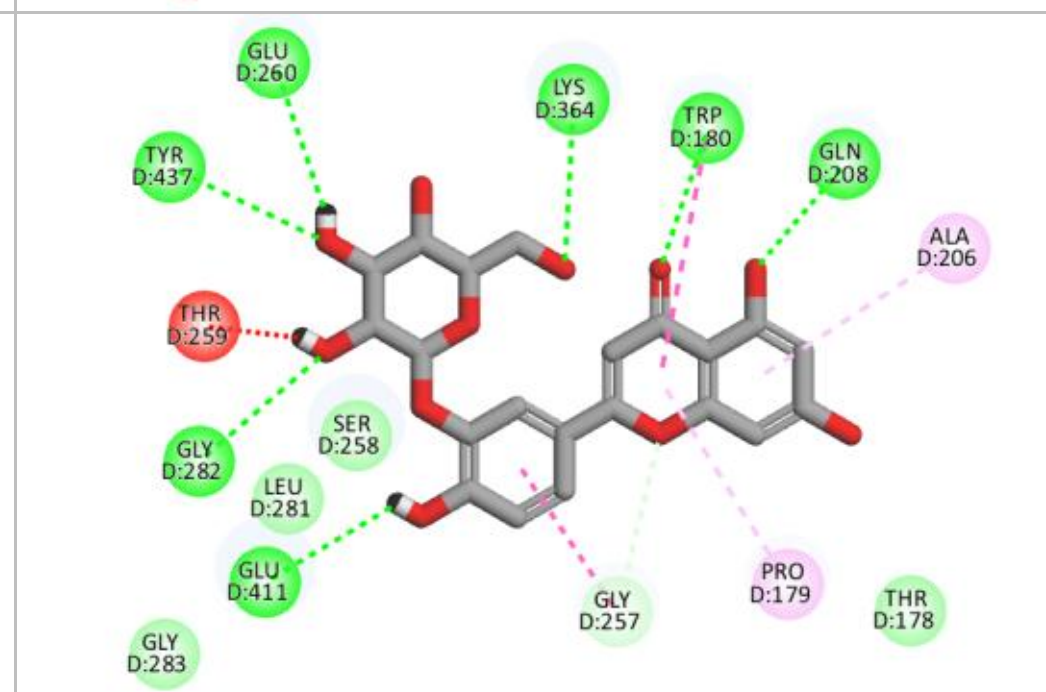</td></tr>
<tr><td>Myricetin</td><td>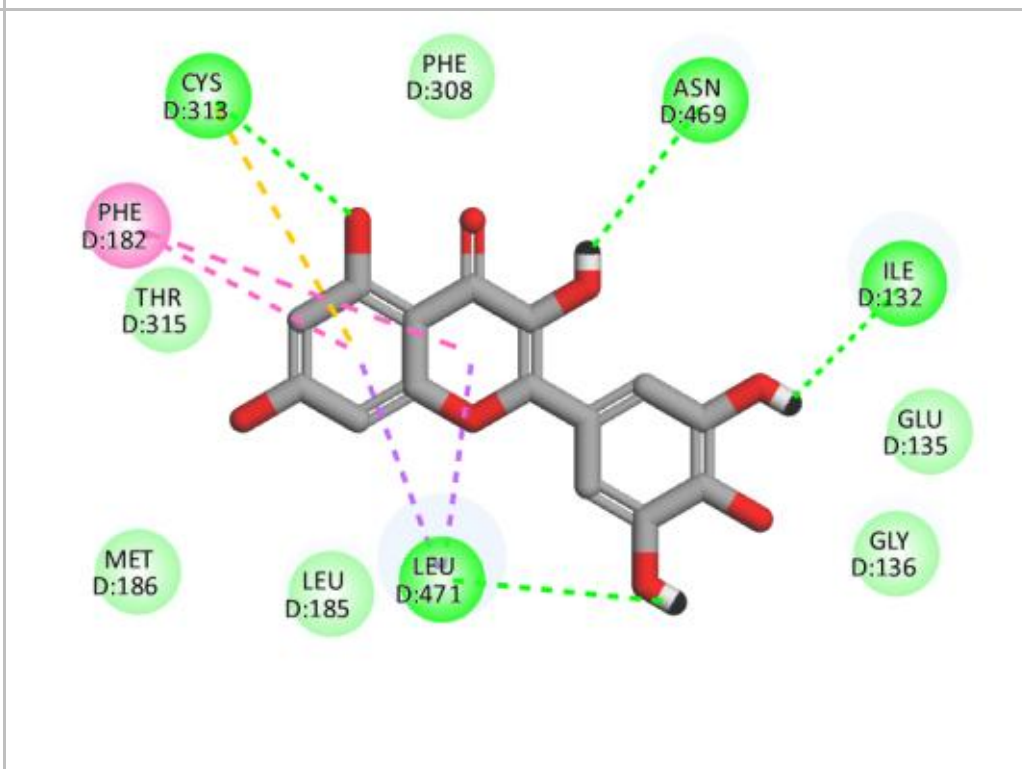</td><td>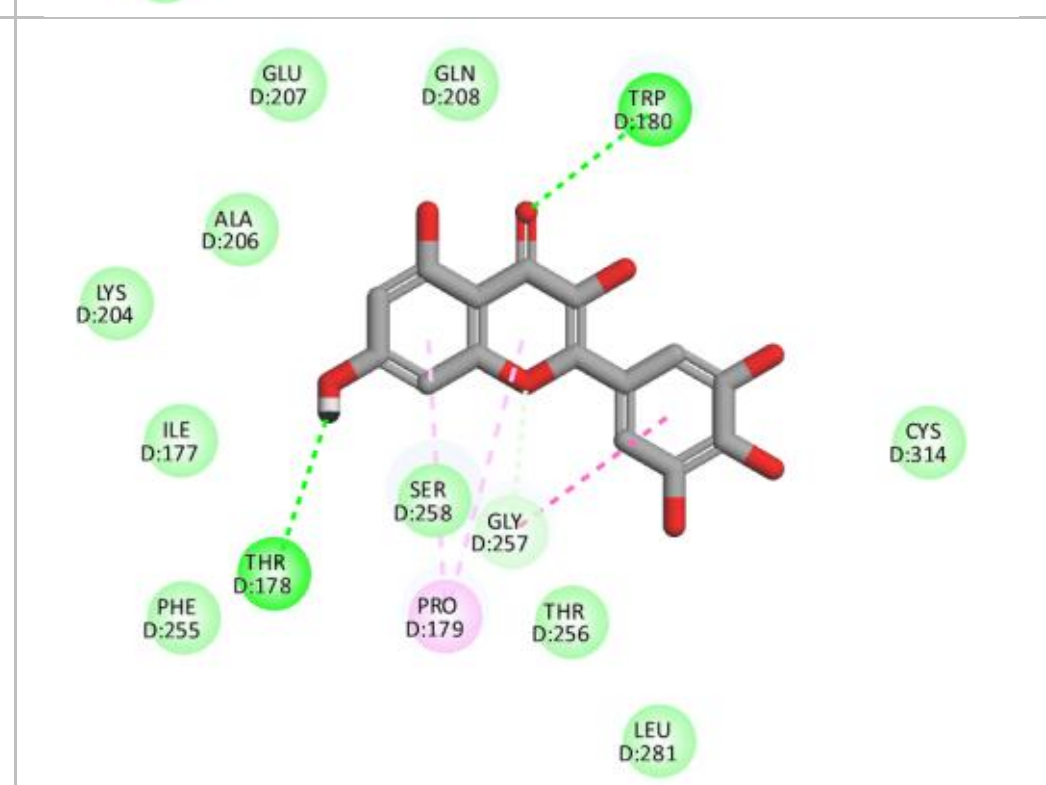</td></tr>
<tr><td>Isovitexin</td><td>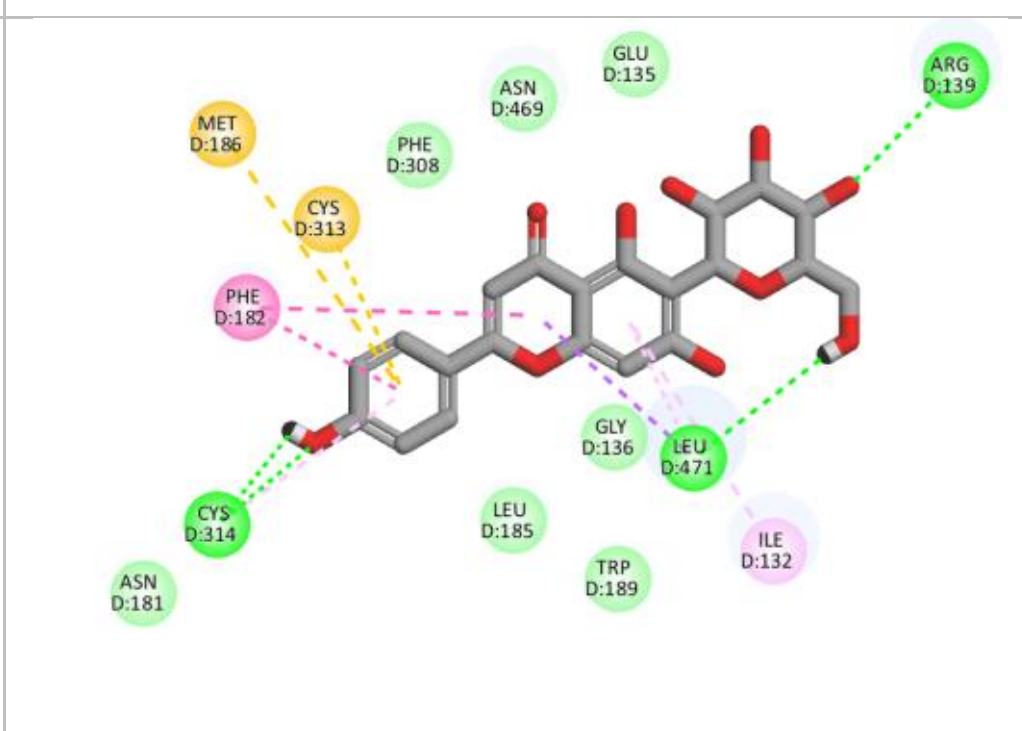</td><td>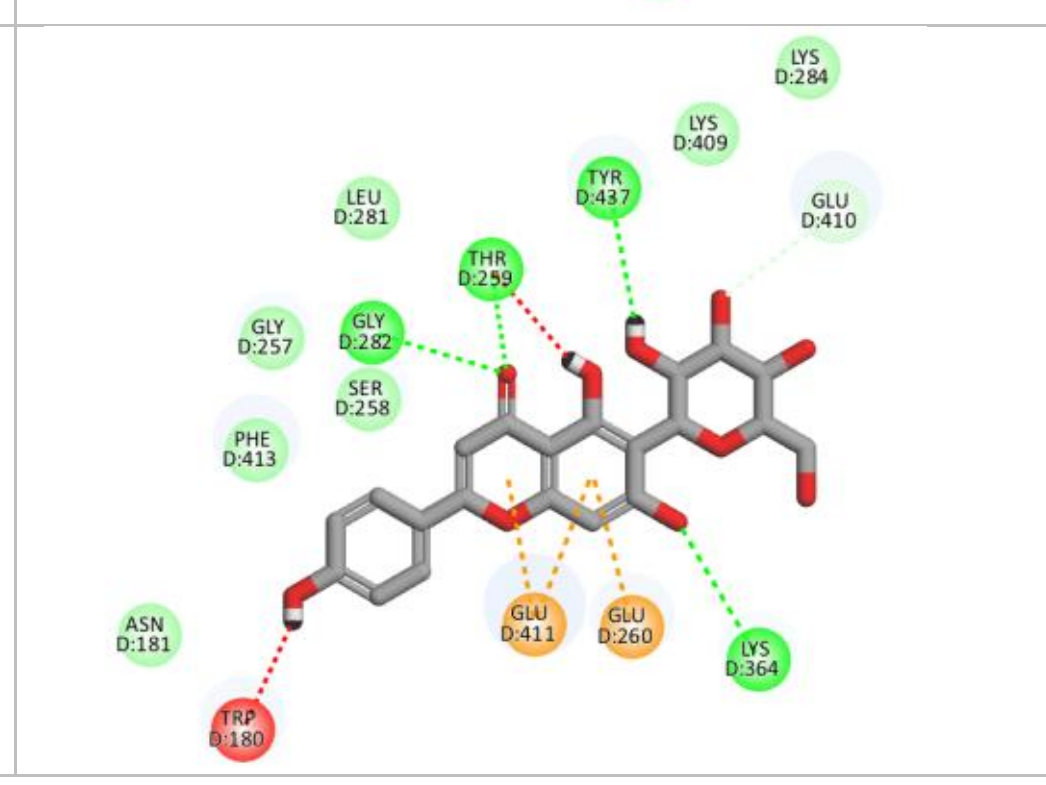</td></tr>
</table>

<table>
<tr><td>Rutin</td><td>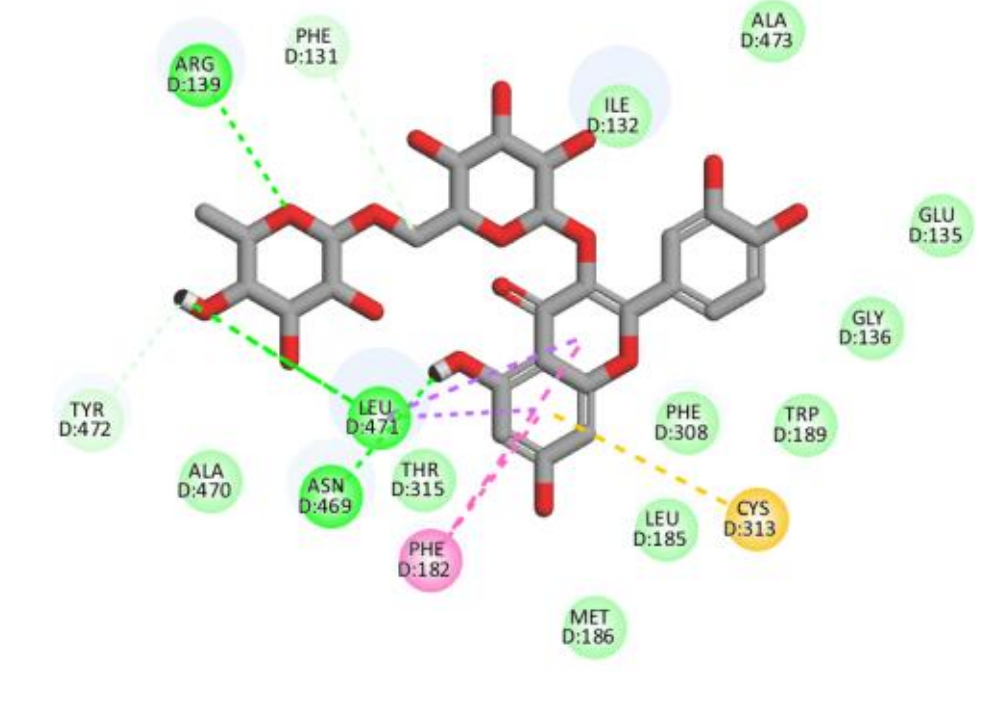
</td><td>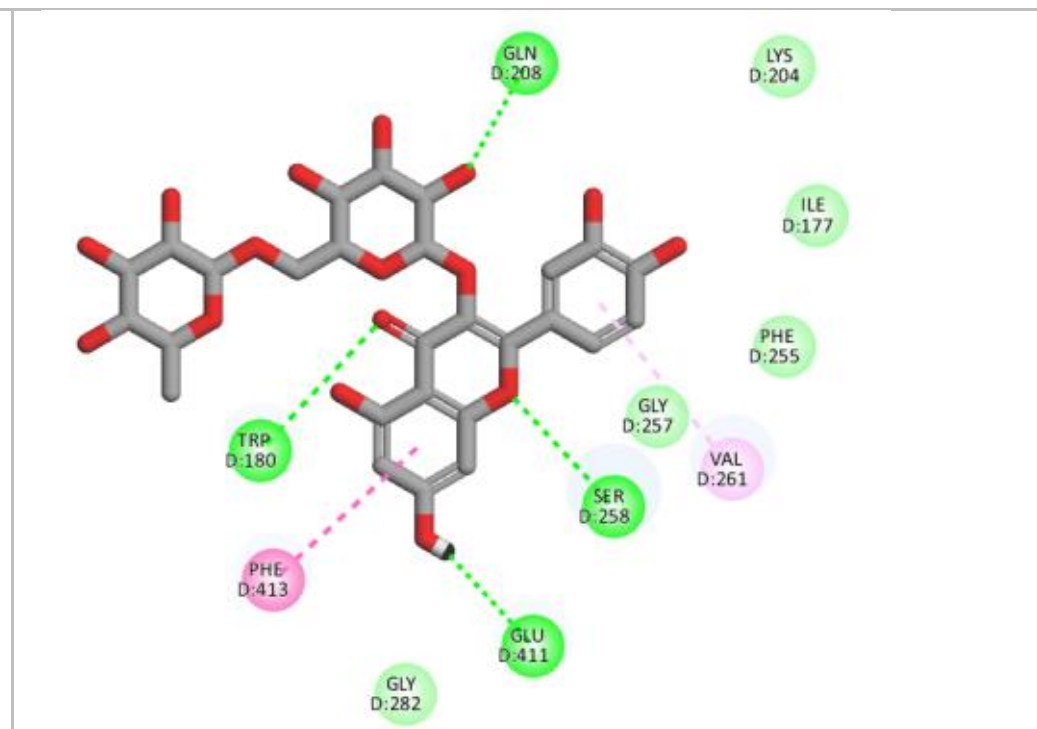
</td></tr>
<tr><td>Luteolin</td><td>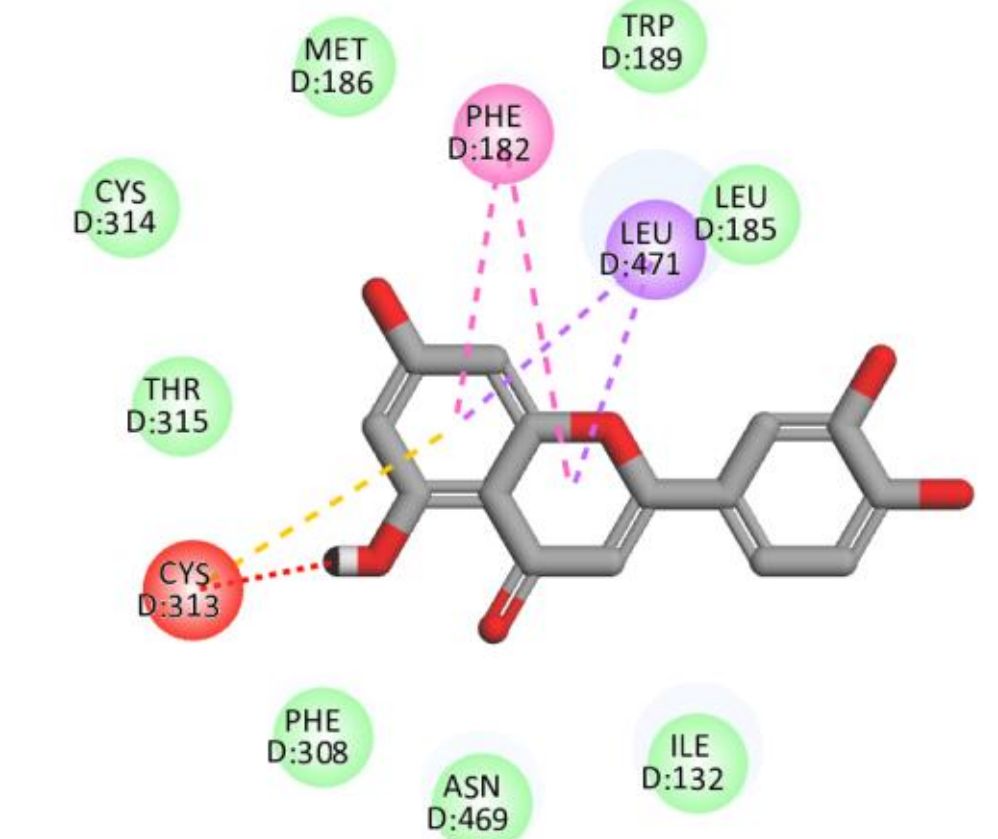
</td><td>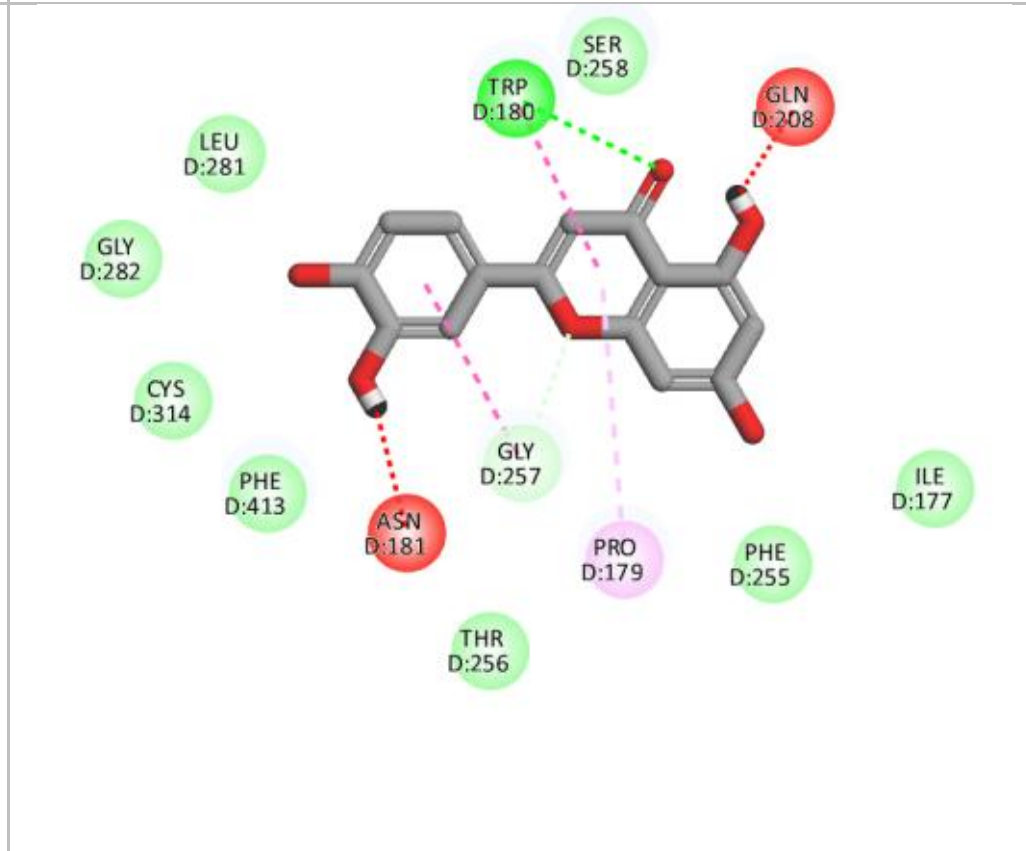
</td></tr>
<tr><td>Quercetin 3-O-galactoside</td><td>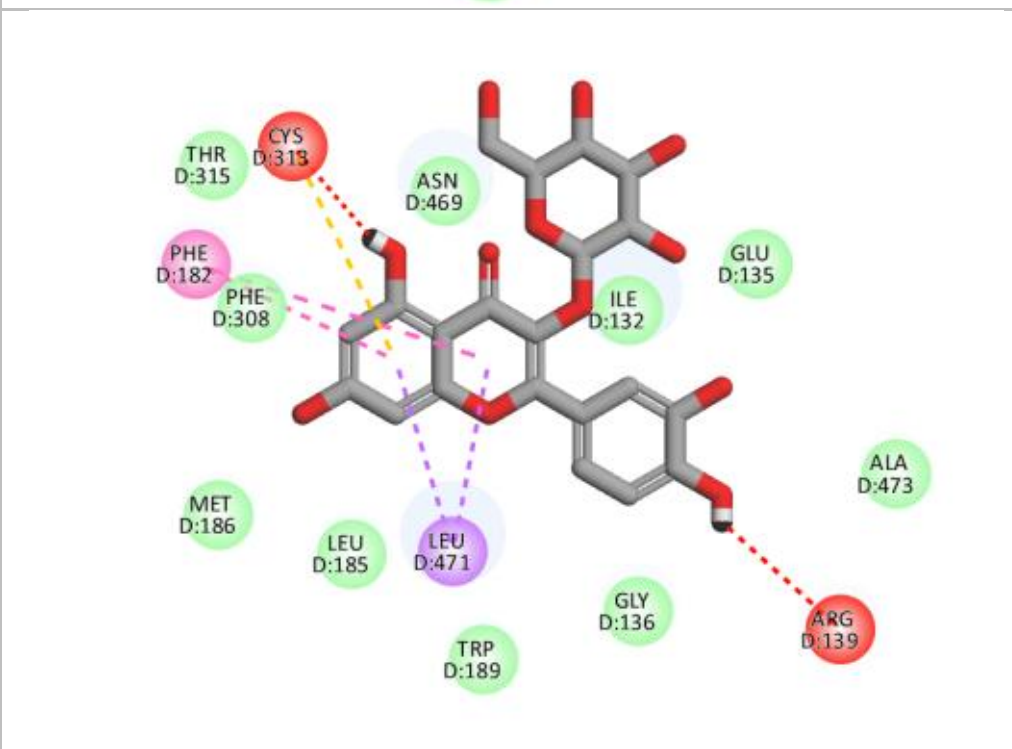
</td><td>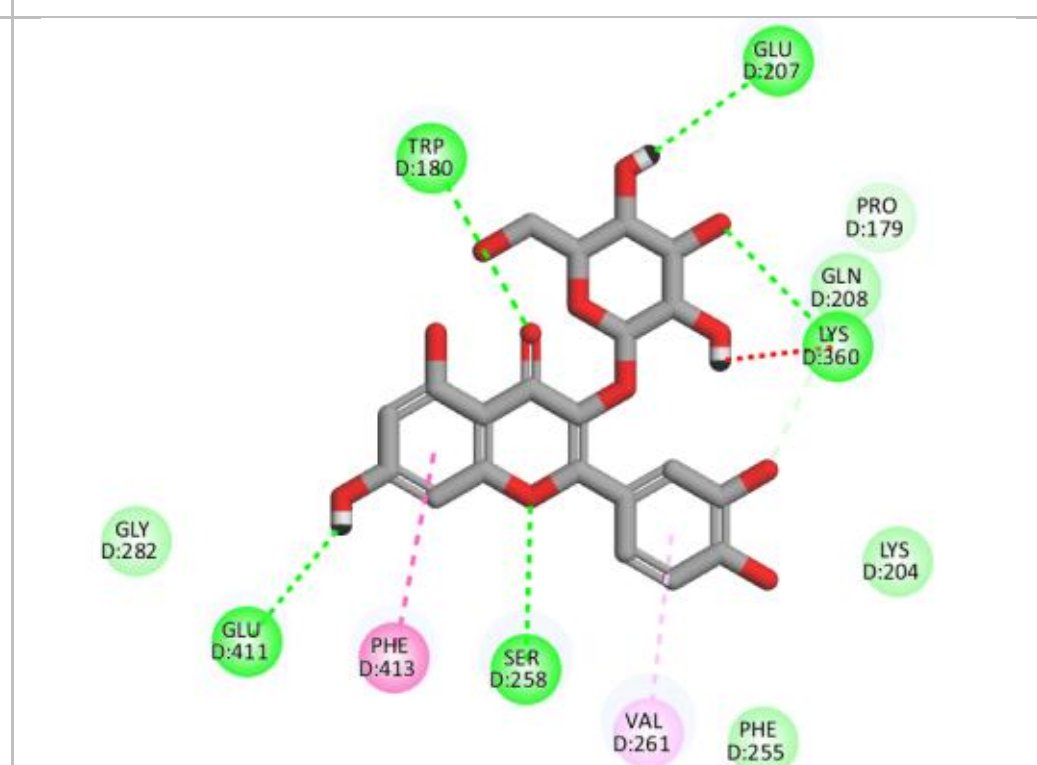
</td></tr>
<tr><td>Chlorogenic Acid</td><td>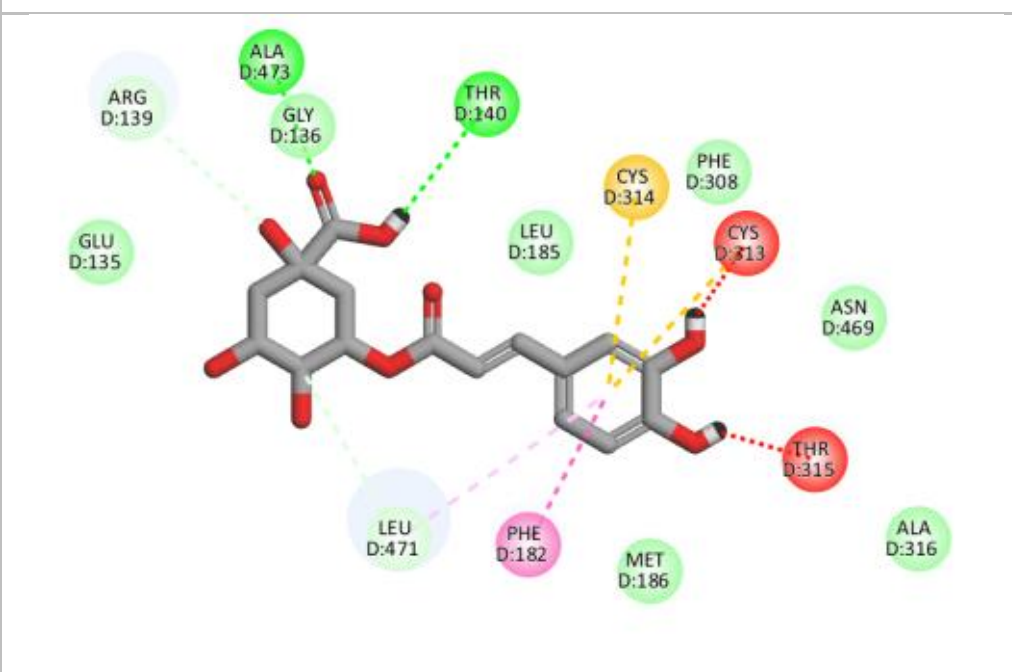
</td><td>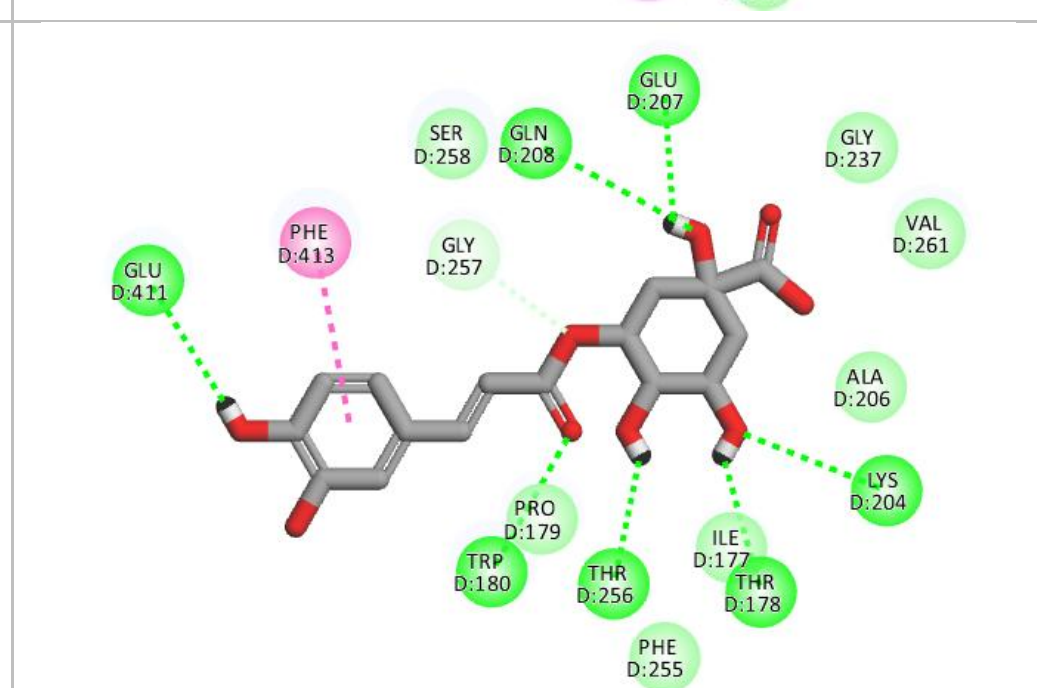
</td></tr>
</table>

5-deoxy luteolin	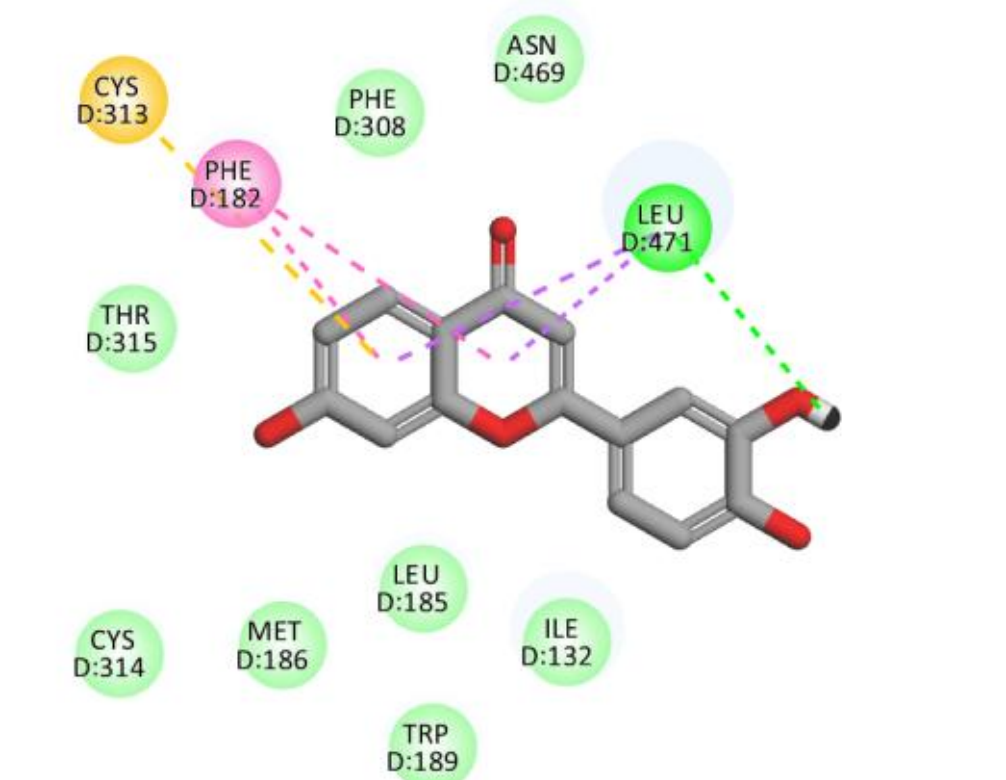	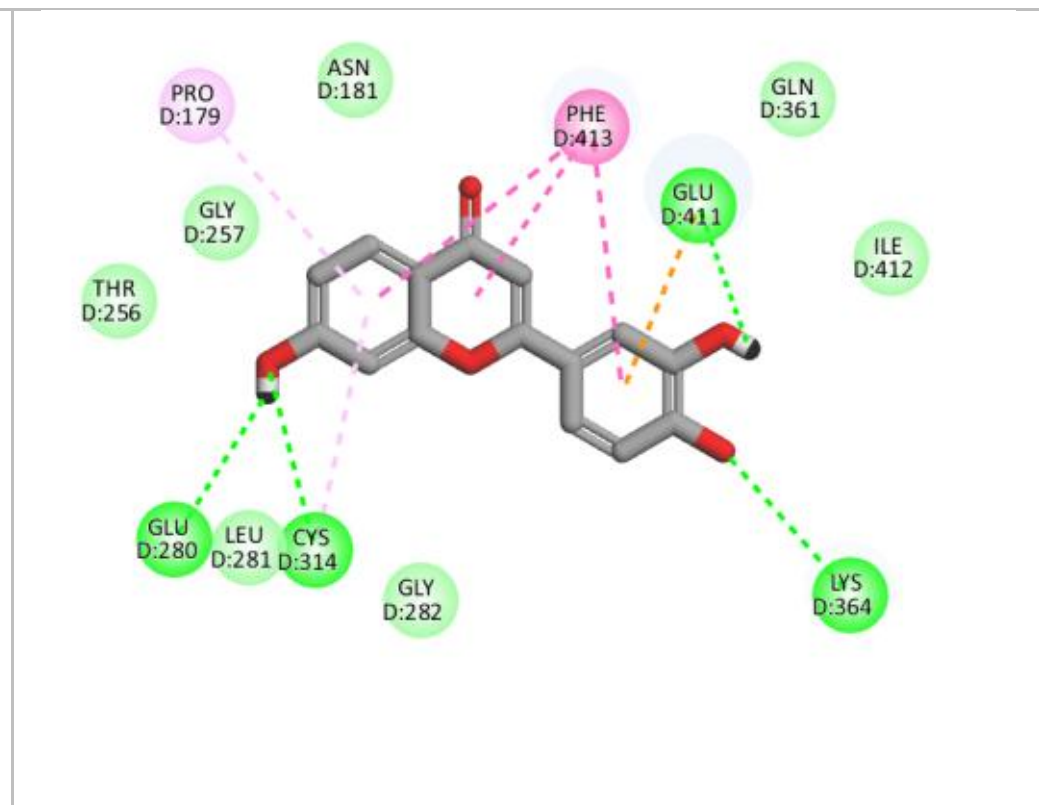
Apigenin	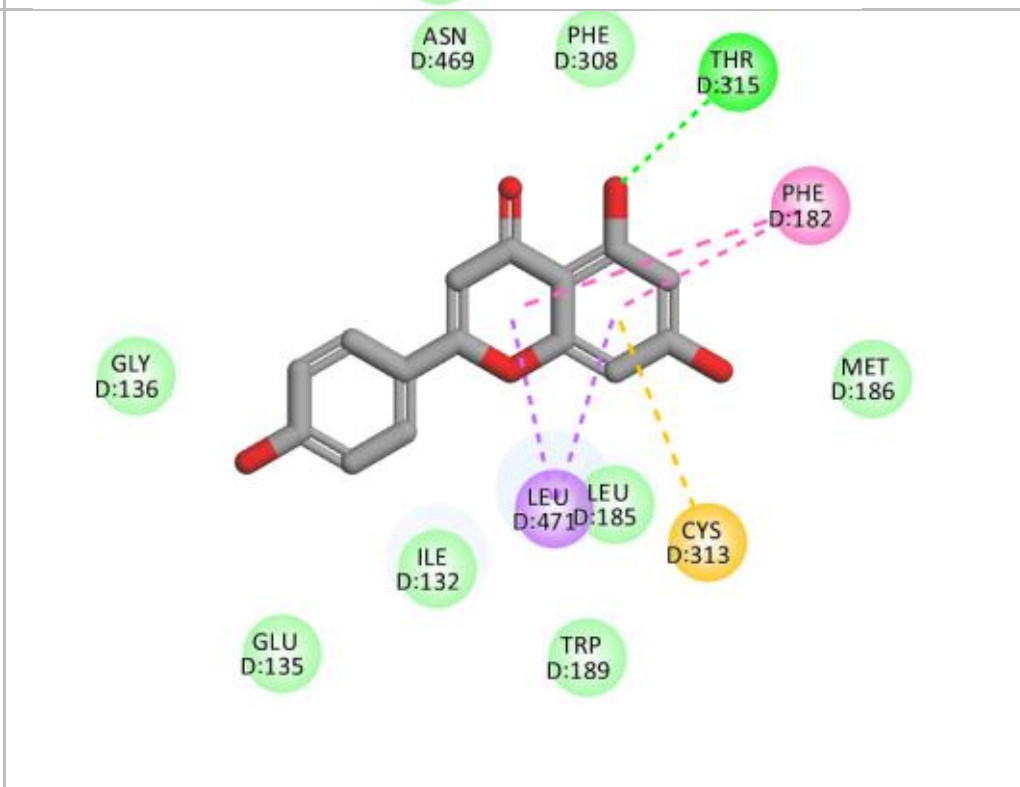	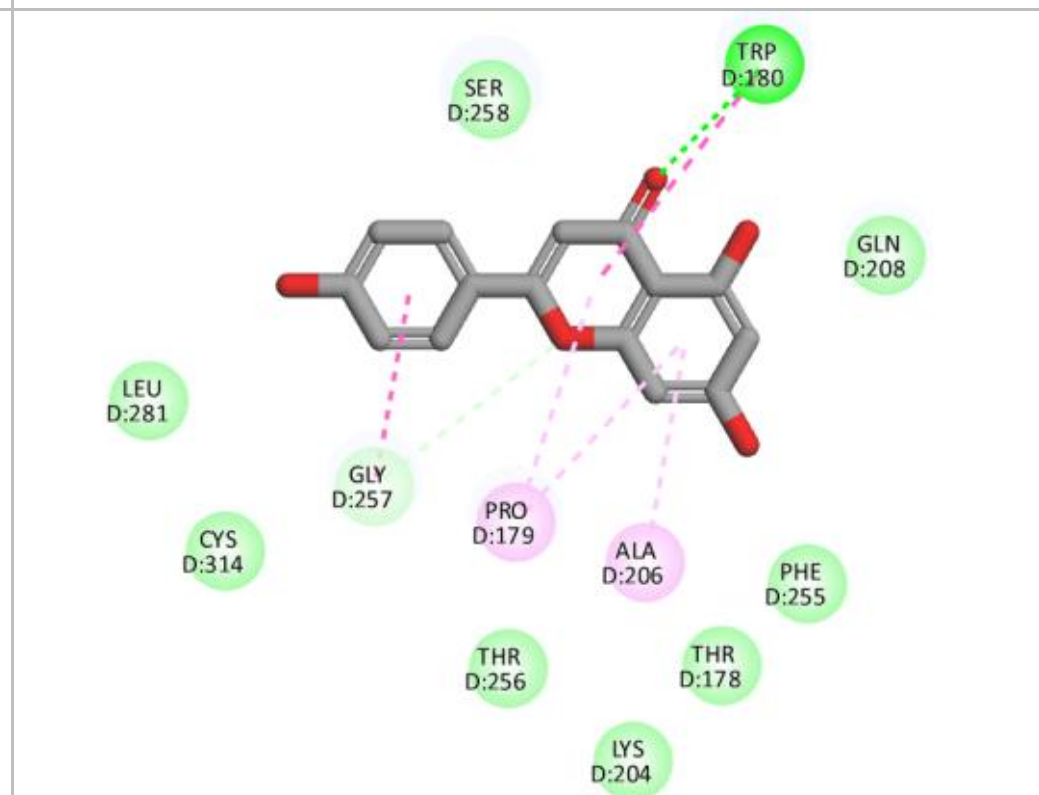
Myricetin 3-O-glucoside	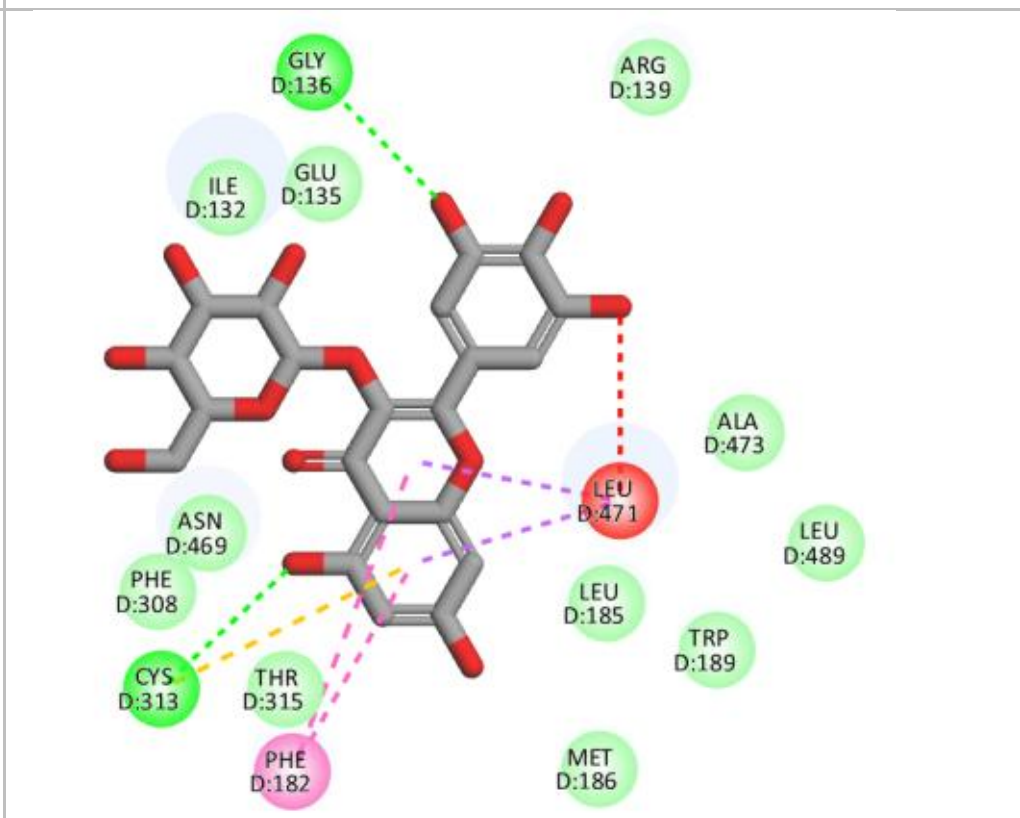	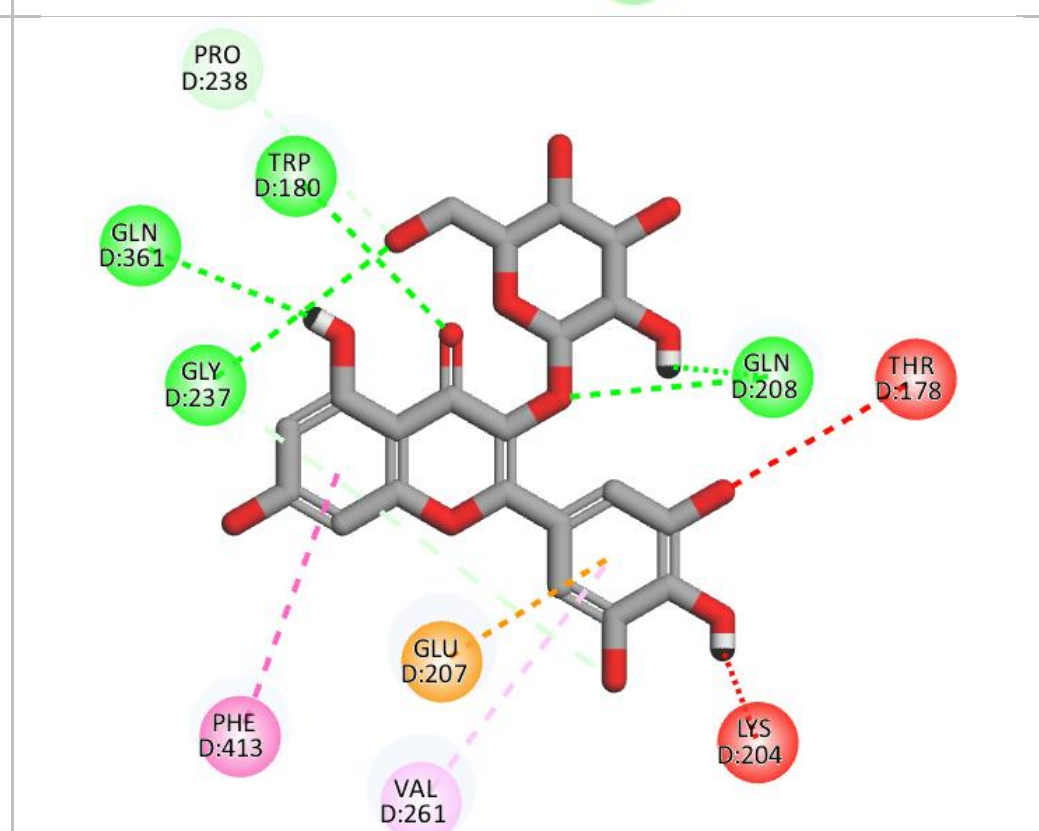
Quercetin 3-methyl ether	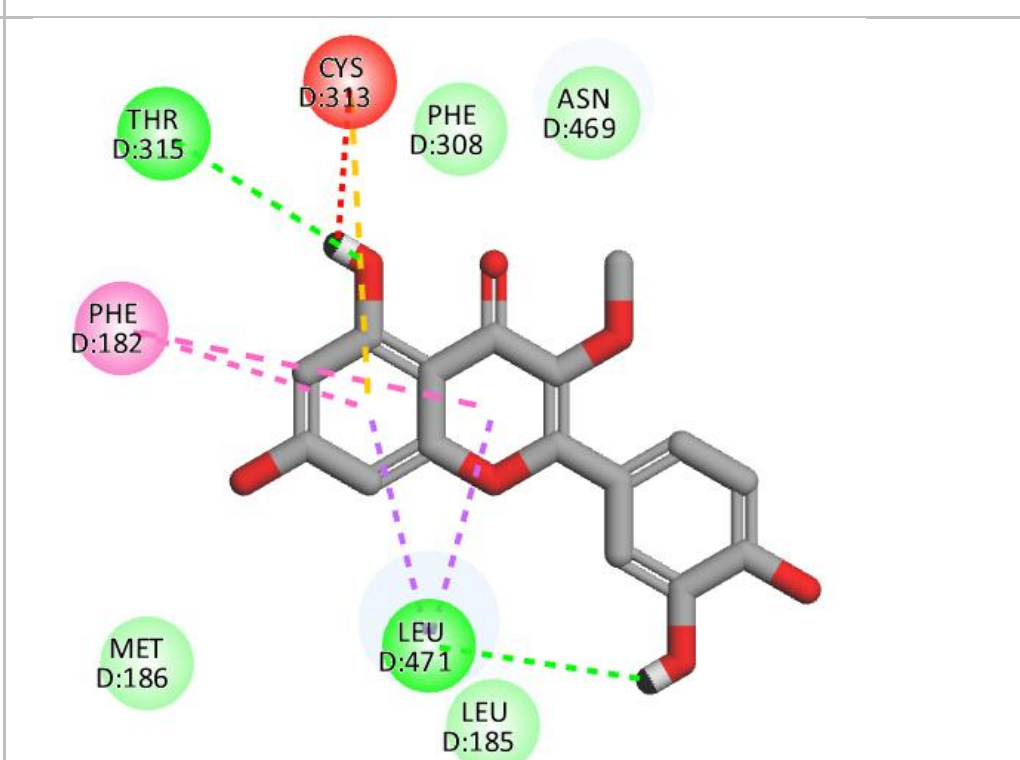	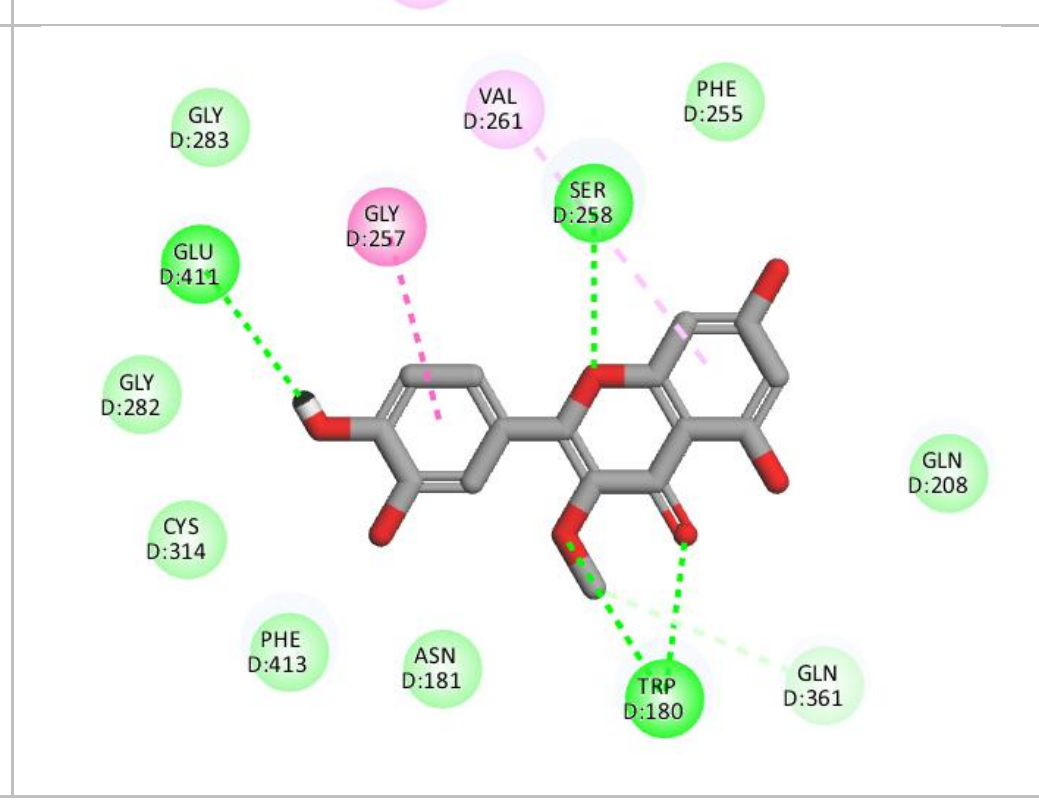

<table>
<tr><td>Vitexin</td><td>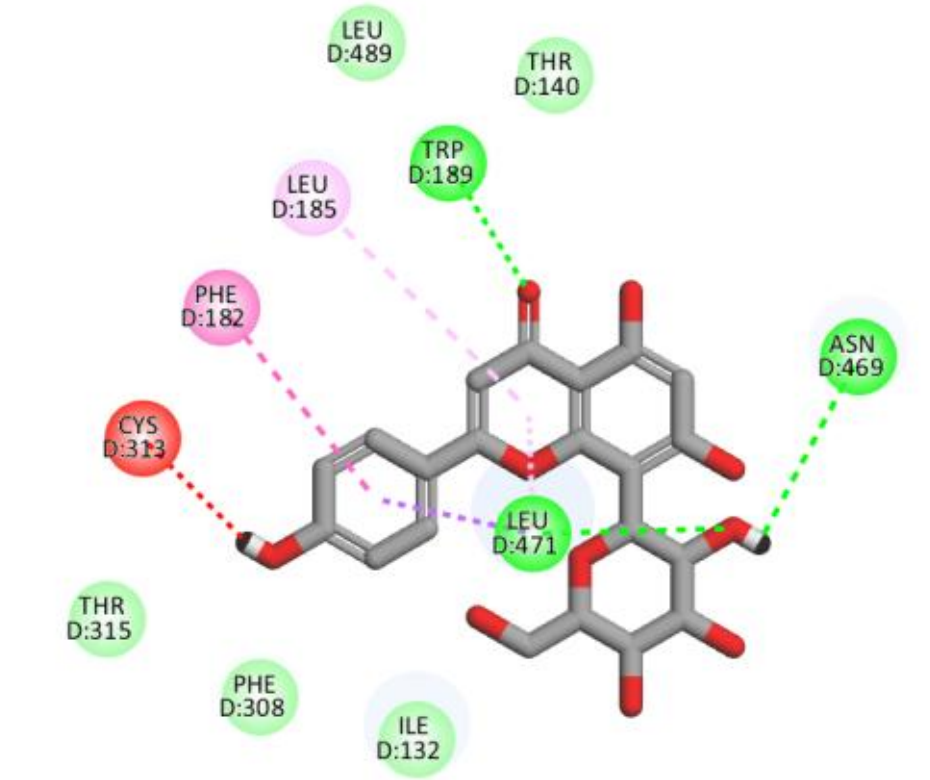</td><td>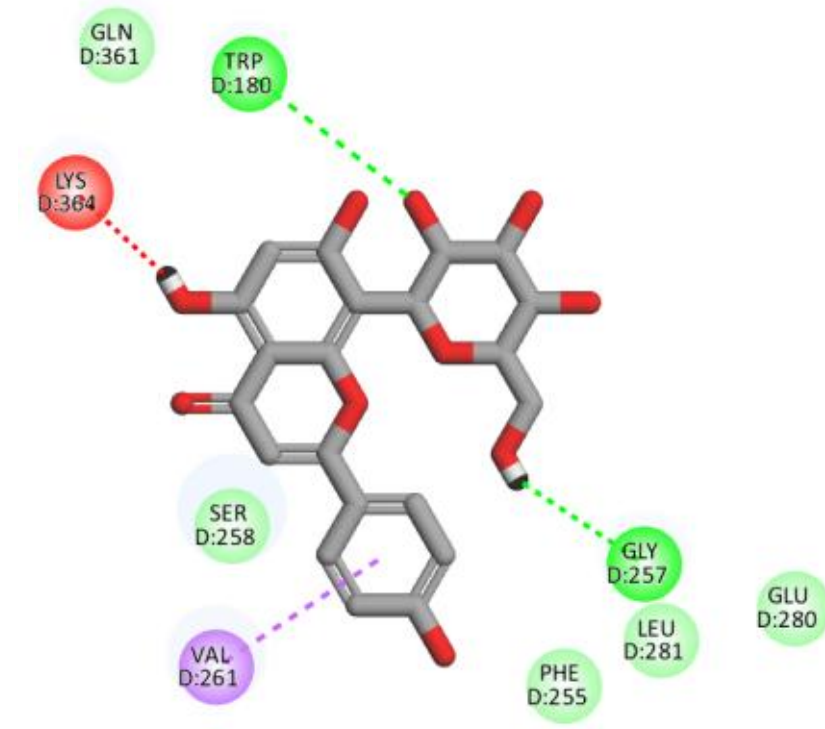</td></tr>
<tr><td>Naringenin</td><td>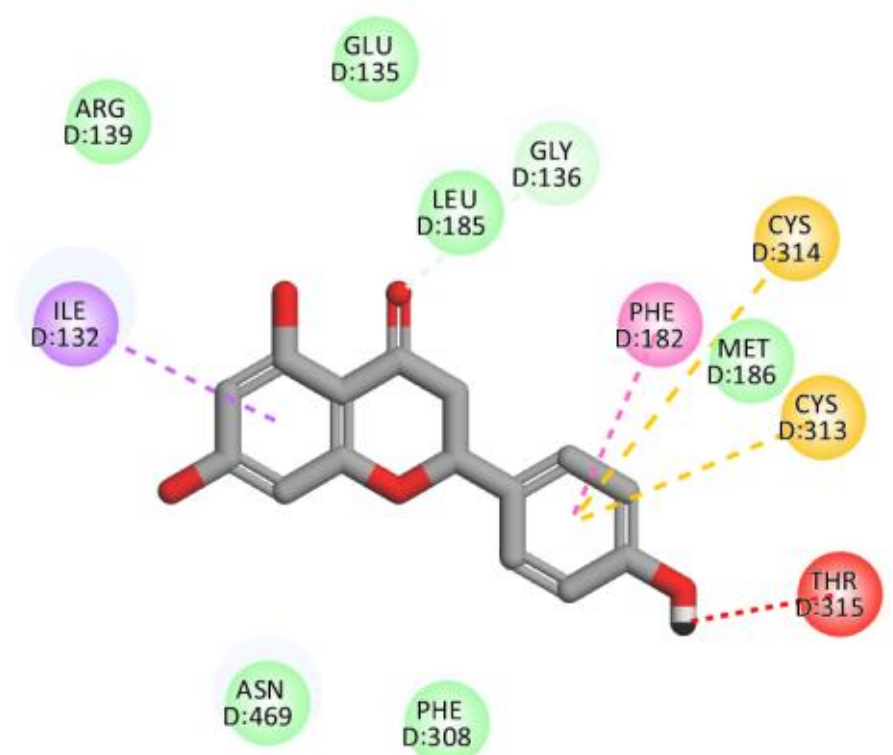</td><td>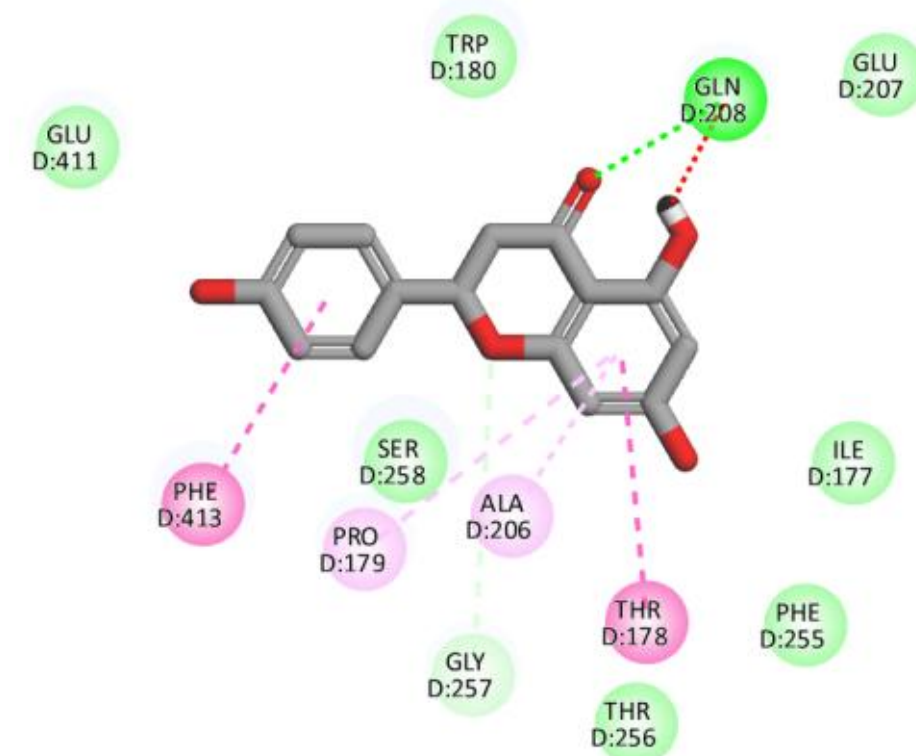</td></tr>
<tr><td>Kaempferol</td><td>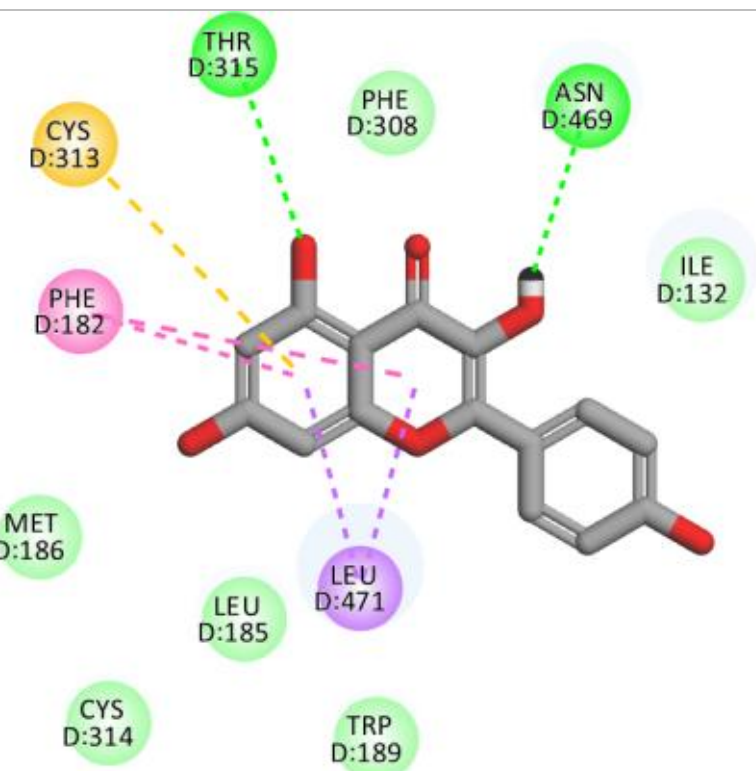</td><td>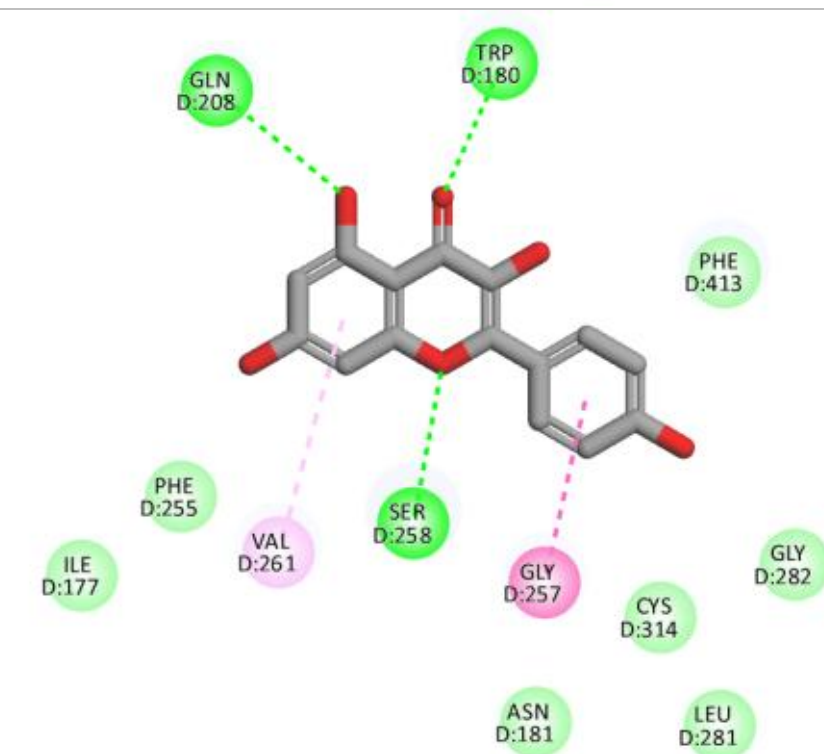</td></tr>
<tr><td>Resveratrol</td><td>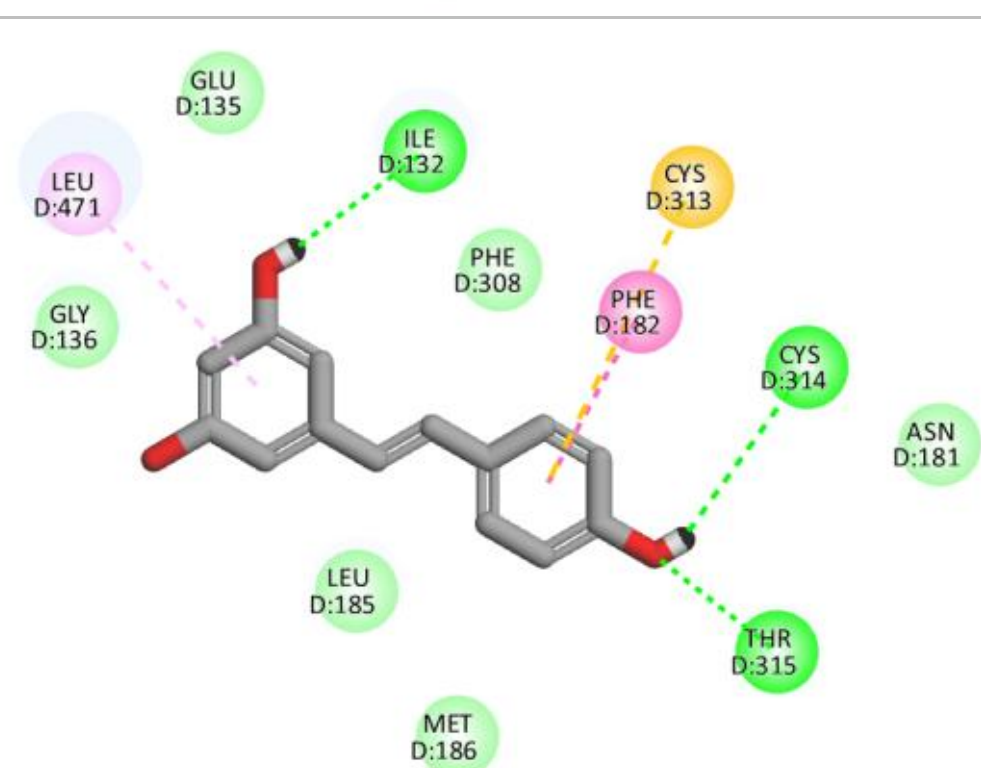</td><td>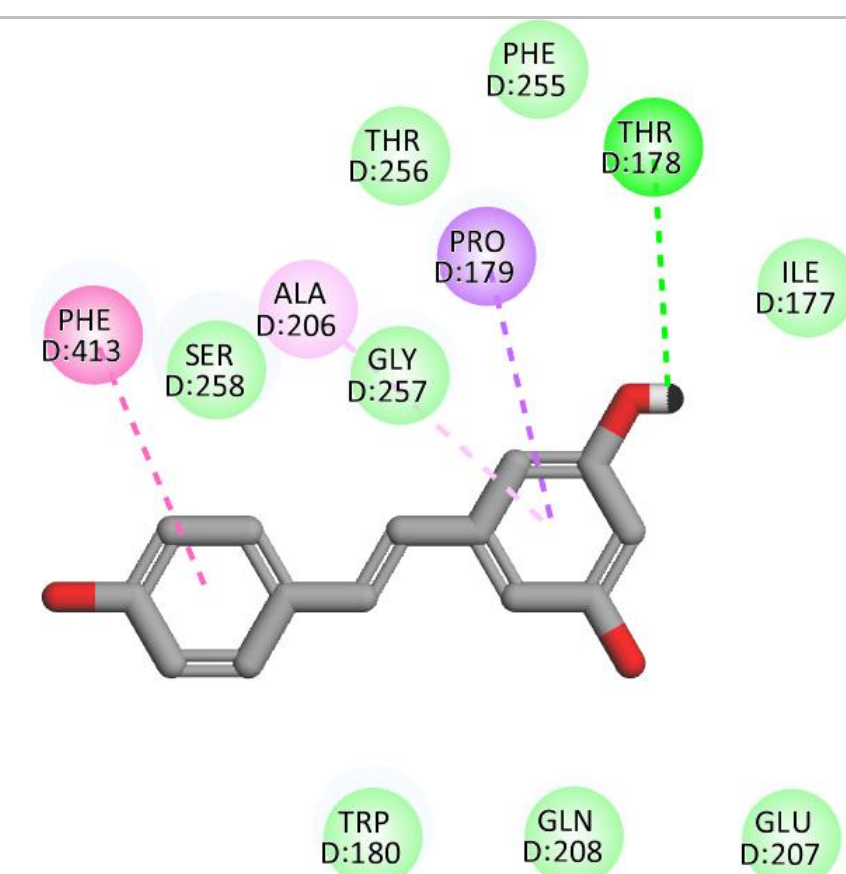</td></tr>
</table>

Quercetin	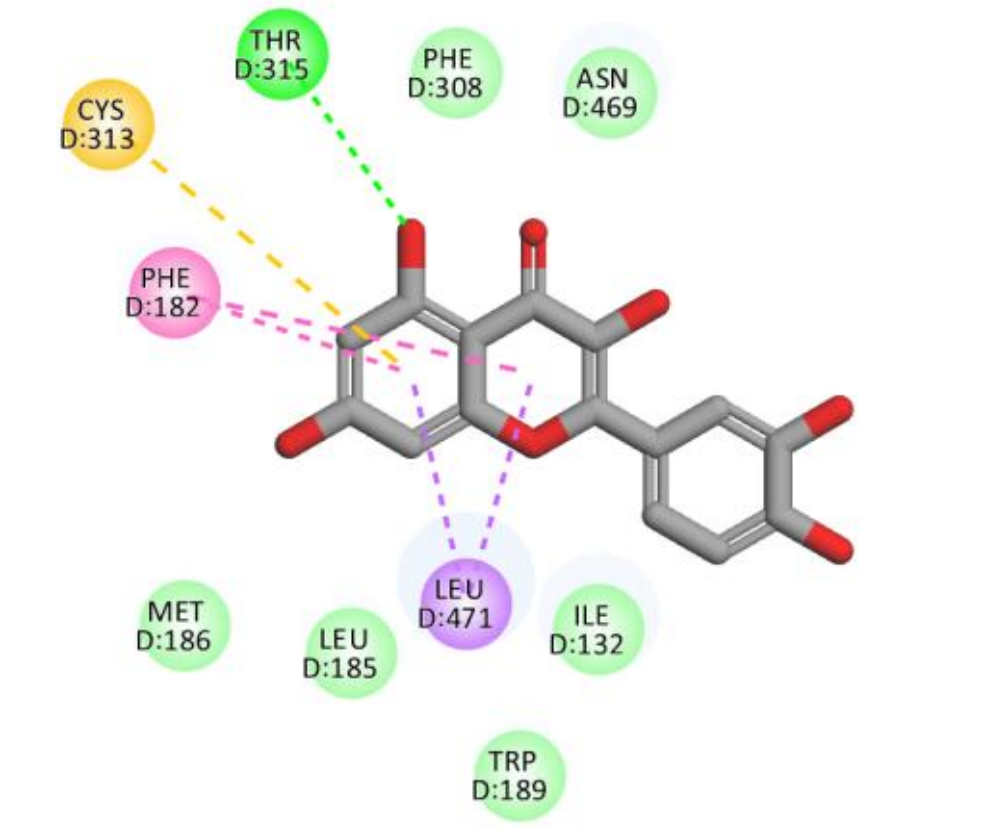	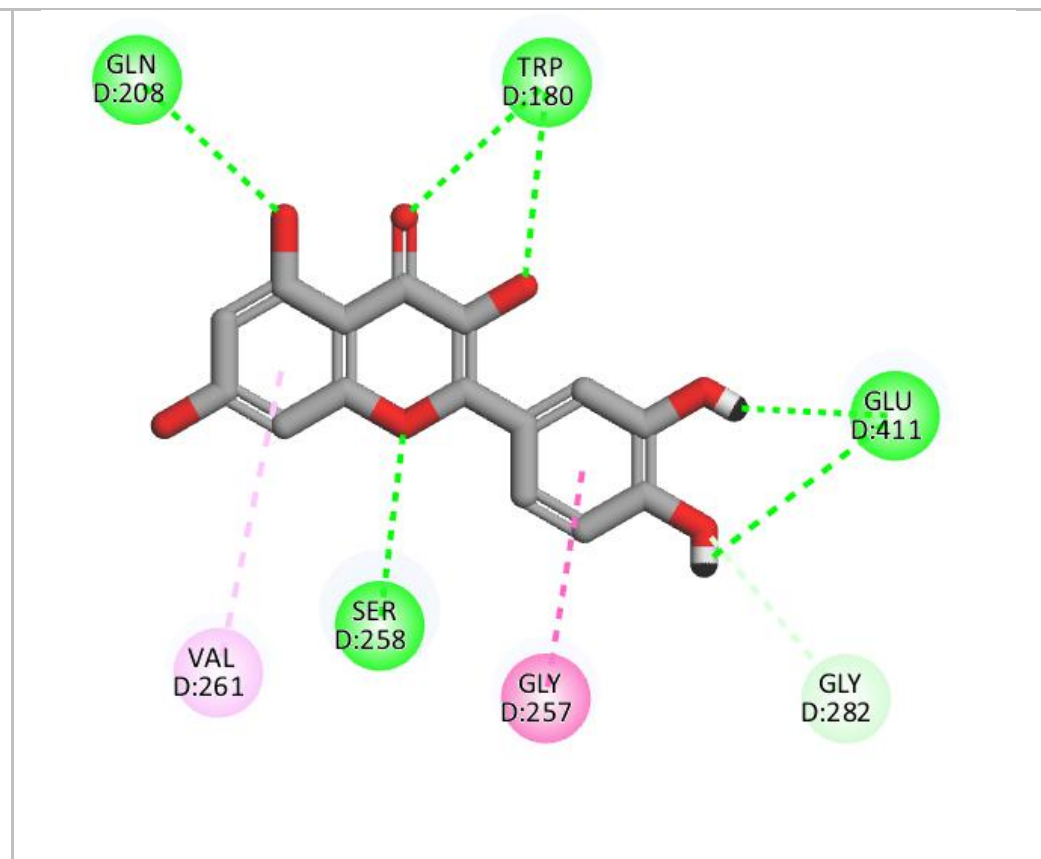
Tannic acid	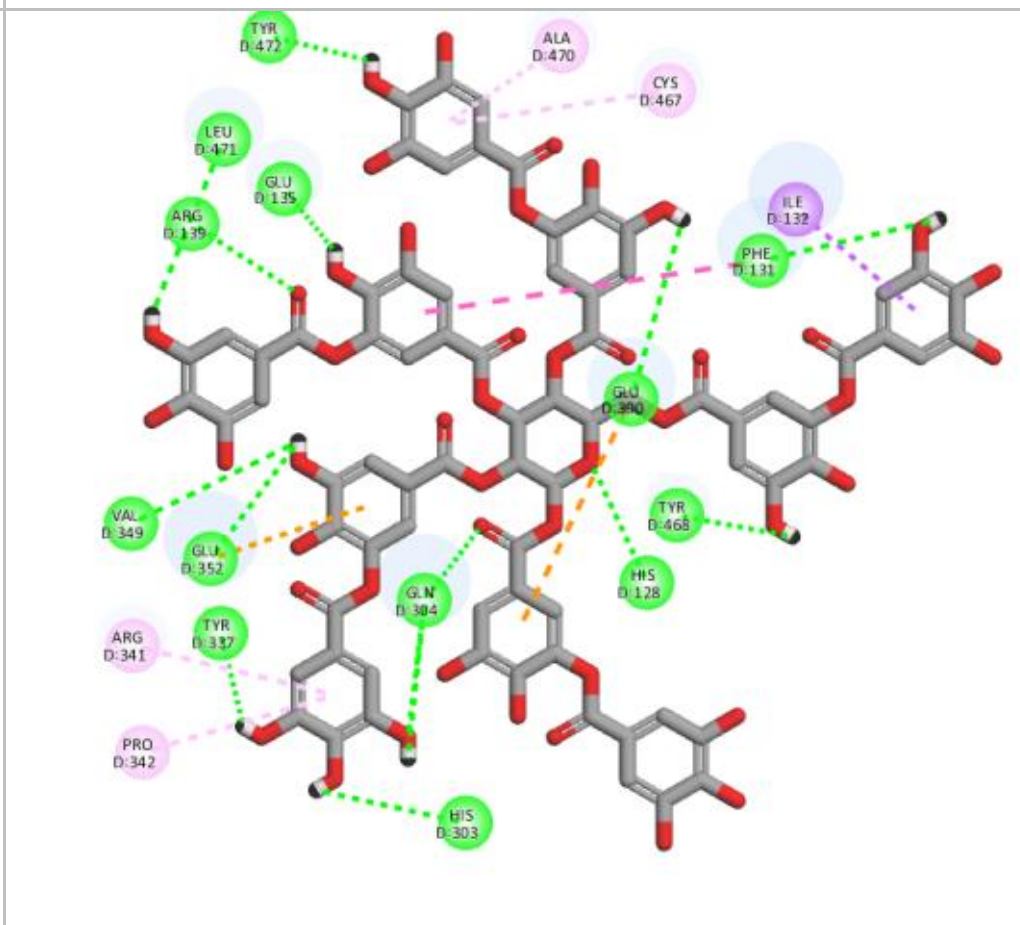	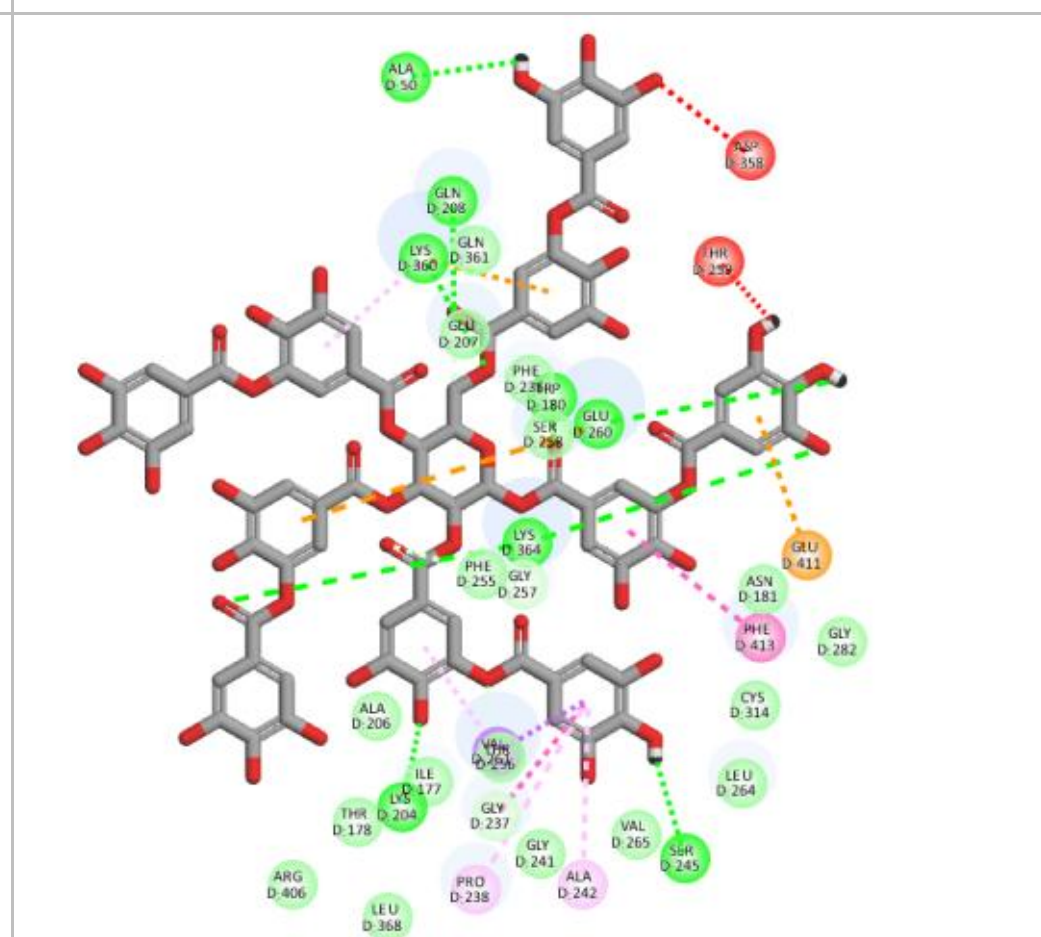
Diadzein	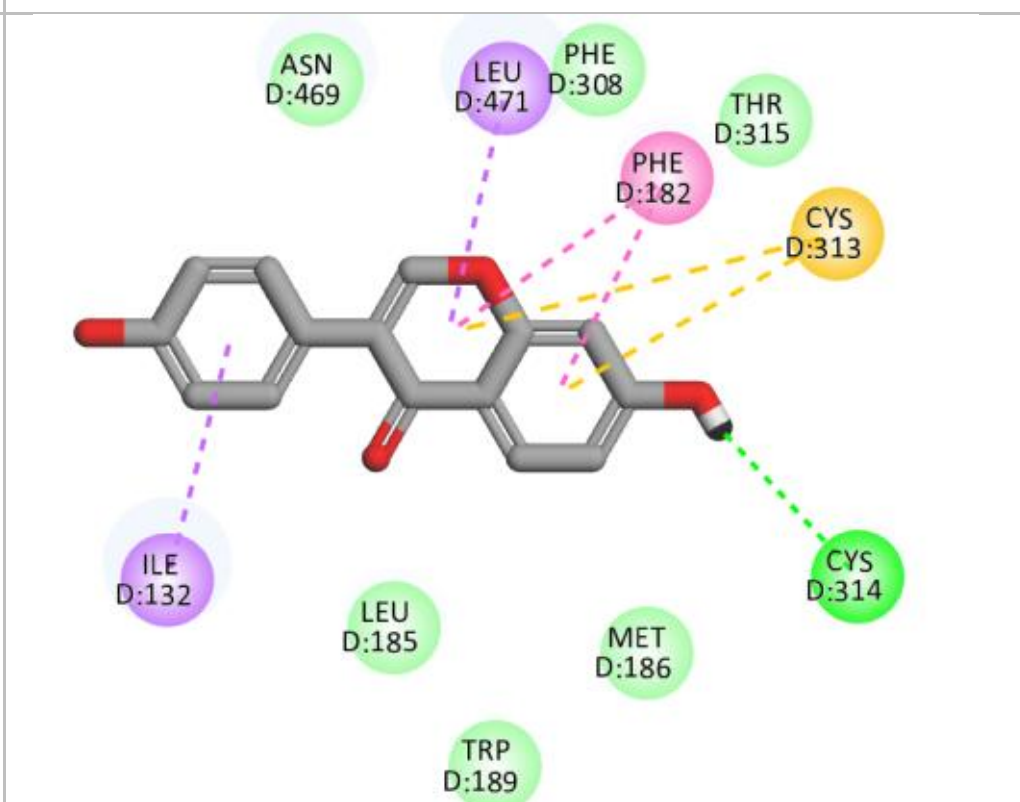	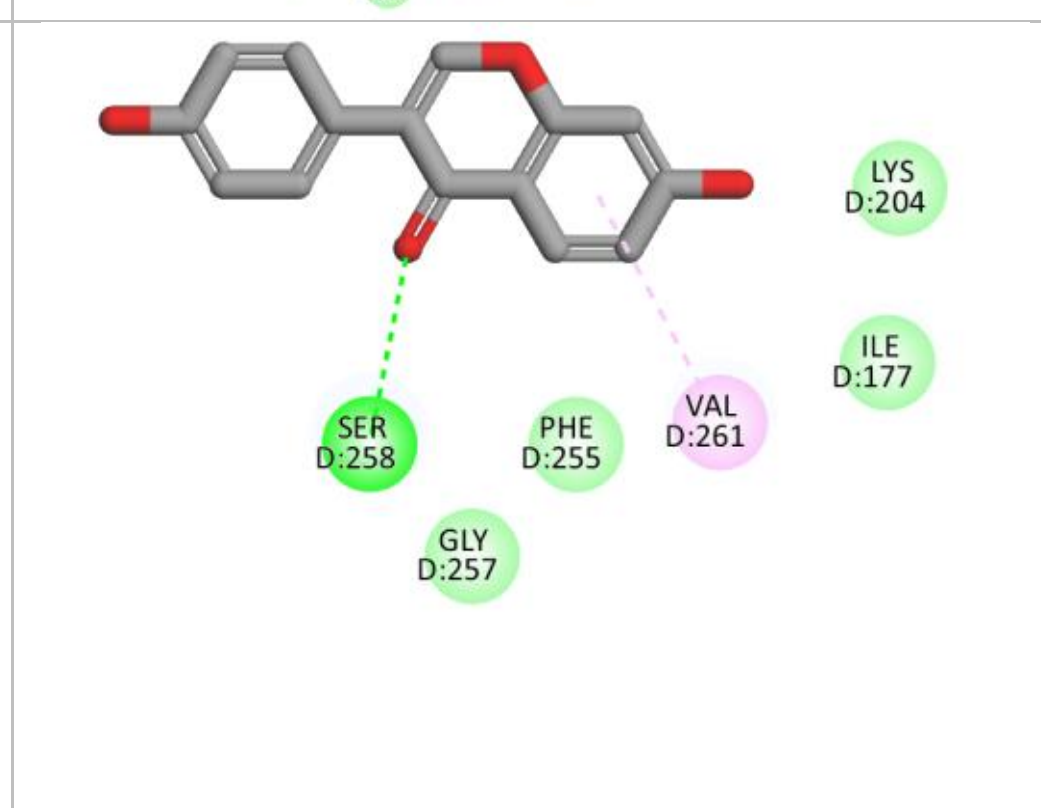
Caffeic acid	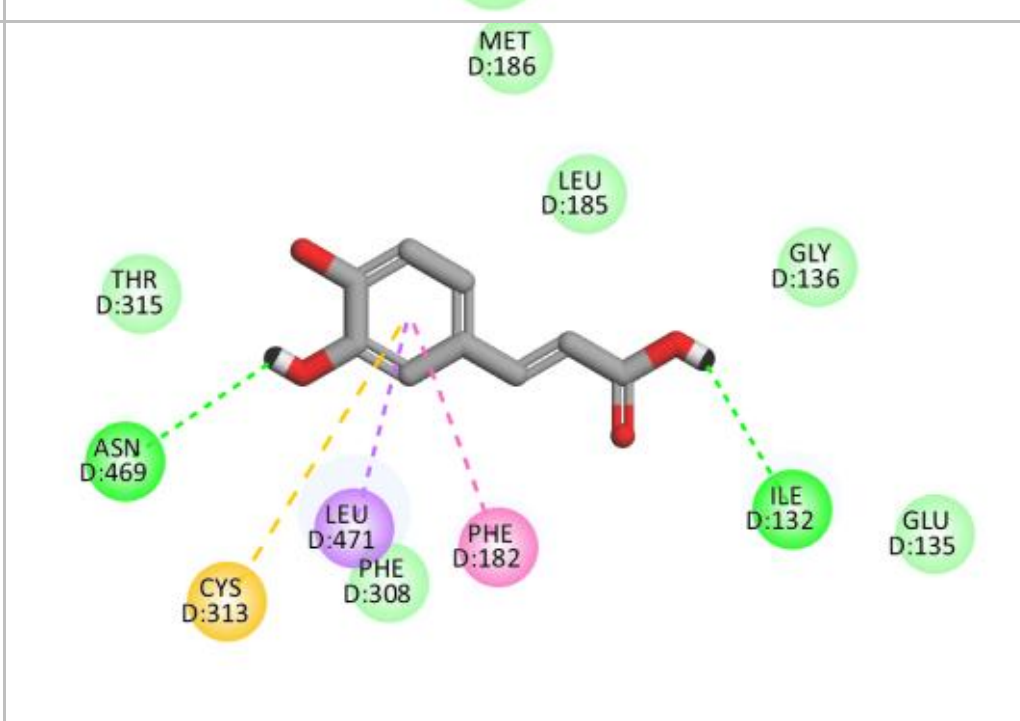	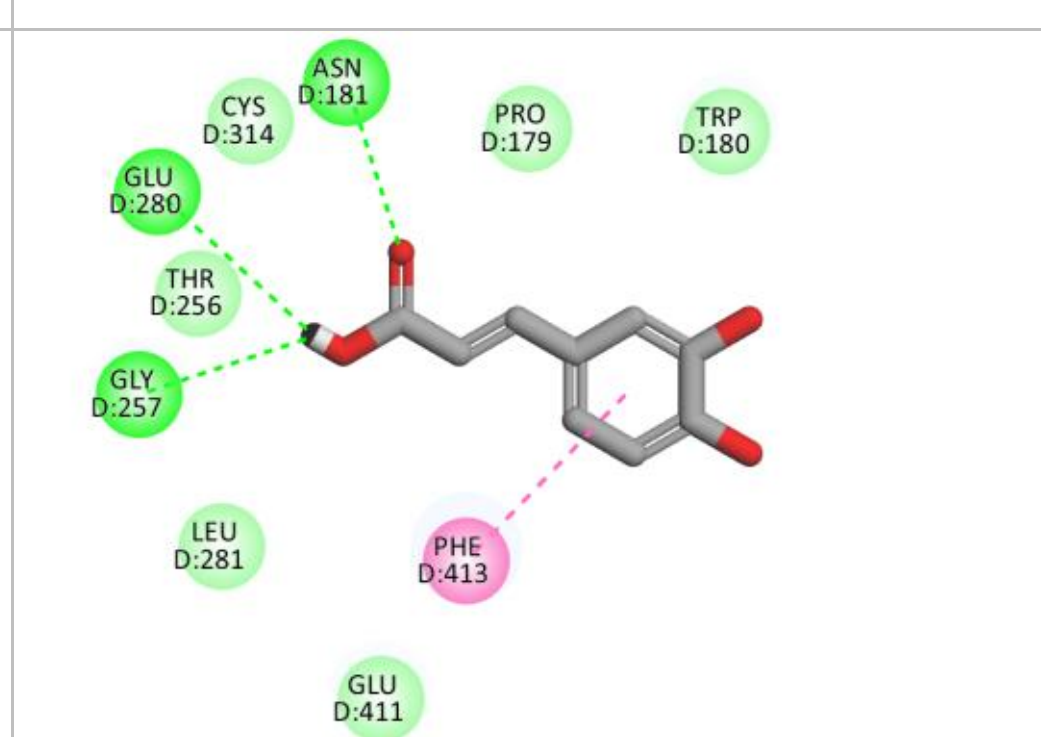

Figure S1. Predicted docking poses of the evaluated phytochemicals within the substrate-binding and cofactor-binding sites of ALDH1A3.

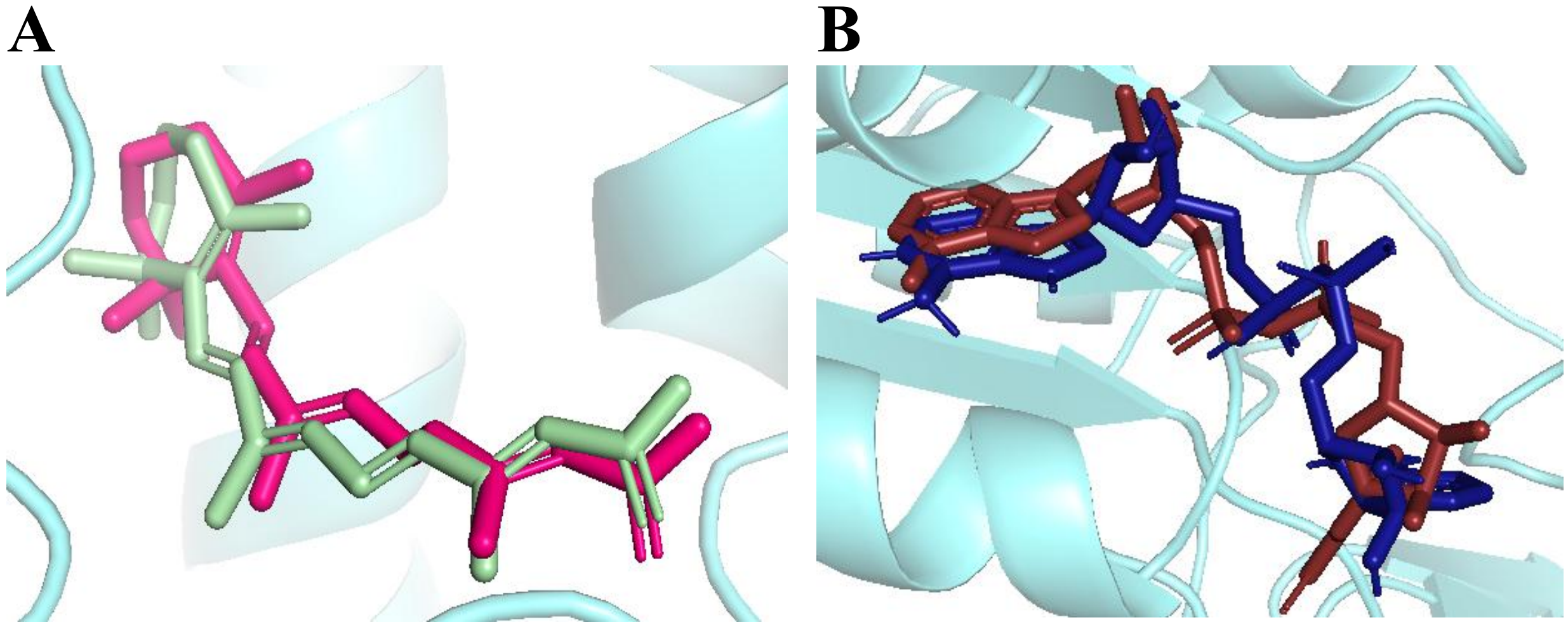

Figure S2. Superimposition of the re-docked and crystallographic ligand poses in ALDH1A3. (A) Re-docked retinoic acid–ALDH1A3 complex (pink) superimposed on the corresponding crystallographic pose (pale green) within the substrate-binding site (RMSD = 0.85 Å). (B) Re-docked NAD–ALDH1A3 complex (navy blue) superimposed on the corresponding crystallographic pose (ruby) within the cofactor-binding site (RMSD = 1.99 Å).

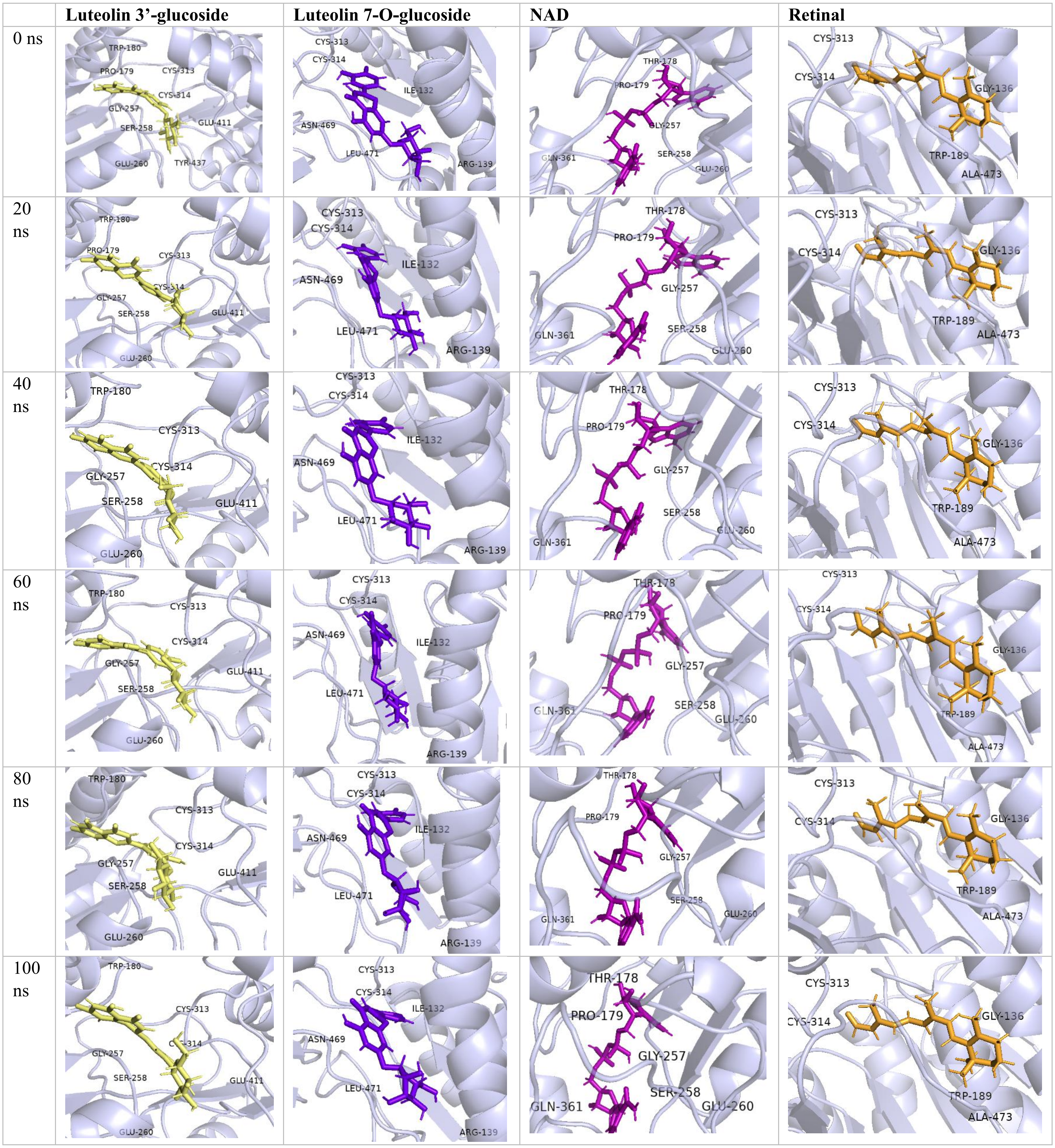

Figure S3. Frames of protein–ligand binding modes every 20 ns for each ligand during simulation.
